# 3-Pyridinols and 5-pyrimidinols: Tailor-made for use in synergistic radical-trapping co-antioxidant systems

**DOI:** 10.3762/bjoc.9.313

**Published:** 2013-12-04

**Authors:** Luca Valgimigli, Daniele Bartolomei, Riccardo Amorati, Evan Haidasz, Jason J Hanthorn, Susheel J Nara, Johan Brinkhorst, Derek A Pratt

**Affiliations:** 1 Department of Chemistry “G. Ciamician”, University of Bologna, Via S. Giacomo 11, I-40126 Bologna, Italy; 2 Department of Chemistry, University of Ottawa, 10 Marie Curie Pvt., Ottawa, Ontario, Canada K1N 6N5

**Keywords:** antioxidants, autoxidation, free radical, phenols, 3-pyridinols, 5-pyrimidinols

## Abstract

The incorporation of nitrogen atoms into the aromatic ring of phenolic compounds has enabled the development of some of the most potent radical-trapping antioxidants ever reported. These compounds, 3-pyridinols and 5-pyrimidinols, have stronger O–H bonds than equivalently substituted phenols, but possess similar reactivities toward autoxidation chain-carrying peroxyl radicals. These attributes suggest that 3-pyridinols and 5-pyrimidinols will be particularly effectiveco-antioxidants when used in combination with more common, but less reactive, phenolic antioxidants such as 2,6-di-*tert*-butyl-4-methylphenol (BHT), which we demonstrate herein. The antioxidants function in a synergistic manner to inhibit autoxidation; taking advantage of the higher reactivity of the 3-pyridinols/5-pyrimidinols to trap peroxyl radicals and using the less reactive phenols to regenerate them from their corresponding aryloxyl radicals. The present investigations were carried out in chlorobenzene and acetonitrile in order to provide some insight into the medium dependence of the synergism and the results, considered with some from our earlier work, prompt a revision of the H-bonding basicity value of acetonitrile to β_2_^H^ of 0.39. Overall, the thermodynamic and kinetic data presented here enable the design of co-antioxidant systems comprising lower loadings of the more expensive 3-pyridinol/5-pyrimidinol antioxidants and higher loadings of the less expensive phenolic antioxidants, but which are equally efficacious as the 3-pyridinol/5-pyrimidinol antioxidants alone at higher loadings.

## Introduction

**Scheme 1:**
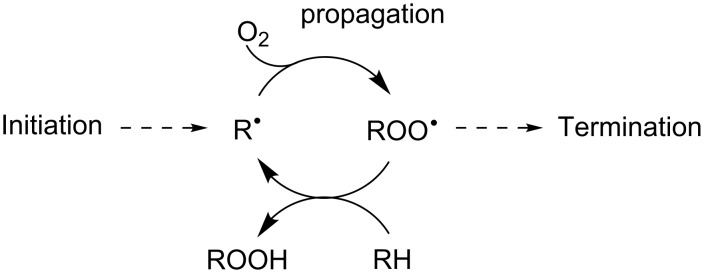
Autoxidation of an organic substrate RH.

**Scheme 2:**
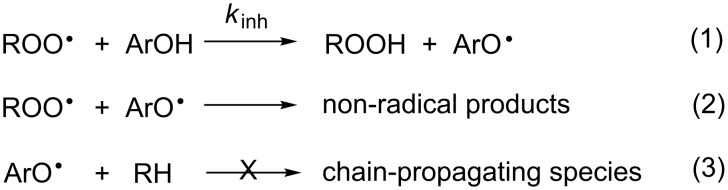
Inhibition of autoxidation by radical-trapping antioxidants (e.g. ArOH).

Radical-trapping (chain-breaking) antioxidants are arguably the most important class of compounds used to protect organic materials from oxidative degradation from autoxidation (Scheme 1) [1–2]. Phenolic compounds are almost universally used for this purpose – for industrial/commercial applications as well as in nature – since they possess inherently high reactivities to chain-carrying peroxyl radicals (ROO^•^) and are readily manipulated to adjust their physical properties for use under specific conditions. The mechanism of the reaction involves the formal transfer of an H-atom from the phenol (ArOH) to a peroxyl radical ROO^•^ (reaction 1 in Scheme 2).

In general, the resultant phenoxyl radical (ArO^•^) is sufficiently unreactive toward the substrate (RH) that it reacts with a second peroxyl radical (reaction 2 in Scheme 2), thereby breaking two oxidative chains per molecule of antioxidant – a ratio commonly referred to as the stoichiometric factor (*n*). However, under some circumstances (e.g. when diffusion of the antioxidant is impeded and it has limited opportunity to encounter other radical species), it is possible for the antioxidant-derived phenoxyl radical to propagate the chain reaction (reaction 3 in Scheme 2). The most relevant example of this is so-called ‘tocopherol mediated peroxidation’ (TMP), which occurs when α-tocopherol (the most biologically active form of vitamin E) is left alone to protect the lipid core of low-density lipoproteins (LDL). LDL is the particle responsible for the distribution of cholesterol in blood plasma and whose oxidation has been linked to the development of cardiovascular disease. Under these conditions, α-tocopherol is not an effective radical-trapping antioxidant [3–4].

For this (and other) reason(s), radical-trapping antioxidants are rarely used alone – be it in nature or industrial/commercial applications. Instead, organic substrates are generally protected from oxidation by the addition of a combination of antioxidants (or co-antioxidants) that function in a synergistic fashion, i.e. they inhibit autoxidation more effectively together than would be expected from the simple additive contributions of their individual antioxidant activities. The interplay of α-tocopherol and ascorbate (vitamin C) in preventing the oxidation of LDL lipids is perhaps the best-known example of such synergism, since the regeneration of α-tocopherol by reduction of the α-tocopheroxyl radical by ascorbate prevents TMP, and effectively turns a water-soluble reducing equivalent into a lipid-soluble one [5–7].

**Scheme 3:**
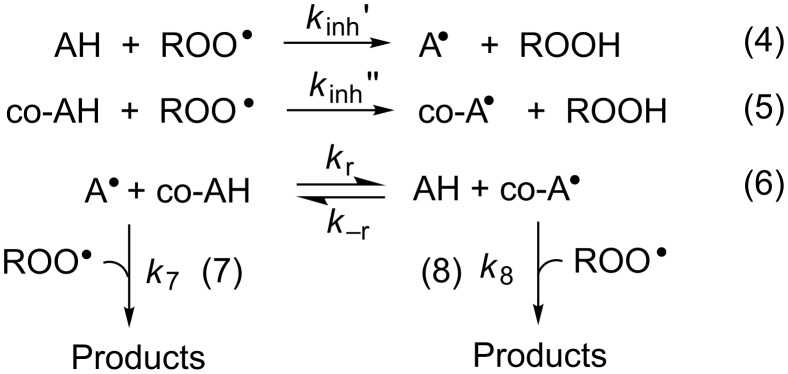
Relevant reactions in co-antioxidant systems.

In recent years, some of us have worked to understand the kinetic and thermodynamic basis for synergism among radical-trapping antioxidants in homogeneous solution, which is summarized in Scheme 3 [8–9]. When two (or more) antioxidants are present in a system, the principal antioxidant (AH) is identified as that which reacts most rapidly with peroxyl radicals than the other(s), the so-called co-antioxidant(s) (co-AH), i.e. *k*_inh_’ > *k*_inh_’’ in reaction 4 and reaction 5 (Scheme 3), respectively. As a result of its greater reactivity, AH must be consumed before co-AH. However, if the equilibrium in reaction 6 (Scheme 3) is favourable, co-AH can regenerate AH for further reaction with ROO^•^. Of course, this is only true if equilibration is faster than consumption of the AH-derived radical by reaction with a second peroxyl radical, i.e. *k*_r_[co-AH] > *k*_7_[ROO^•^] for reaction 6 and reaction 7 (Scheme 3), respectively. However, this is a condition that is generally easily met since [ROO^•^]_ss_ in the presence of AH/co-AH must be very low and *k*_r_ for phenol/phenoxyl couples is normally ≥10^4^ M^−1^s^−1^ [8–9].

Based on this model, in order for synergism to occur among equilibrating phenolic antioxidants it is necessary that the principal antioxidant has both a higher reactivity with peroxyl radicals (*k*_inh_) and a higher O–H bond dissociation enthalpy (BDE), as compared to the co-antioxidant. Unfortunately, this is a very demanding requirement since *k*_inh_ and the O–H BDE are inversely correlated according to well-established Evans–Polanyi relationships [2,10].

**Figure 1:**
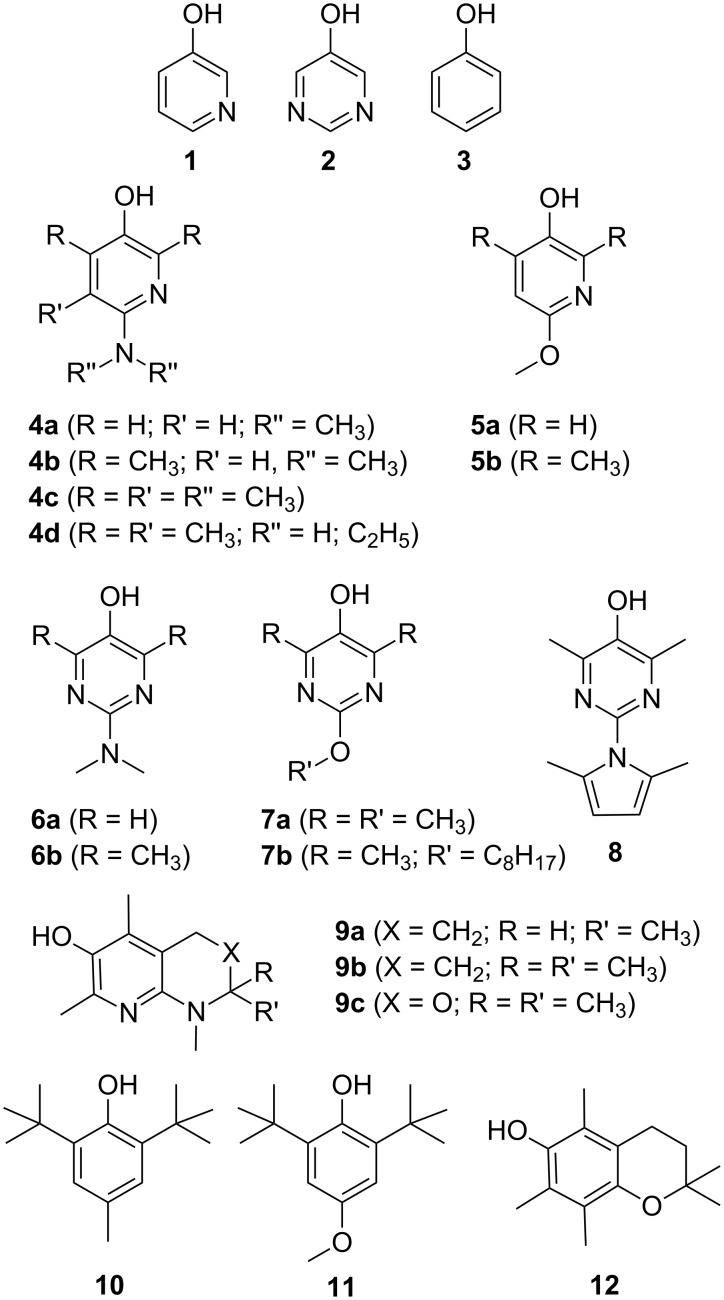
Relevant structures **1**–**12**.

Over the years, our research groups have developed novel air-stable and highly reactive radical-trapping chain-breaking antioxidants based on either 3-pyridinol (**1**) or 5-pyrimidinol (**2**) core structures (Figure 1) [11–17]. Compared to equivalently-substituted phenols, these compounds have been shown to possess stronger O–H bonds (e.g. +1.4 kcal/mol for **1** and +2.5 kcal/mol for **2** relative to **3**) while maintaining similar or higher reactivity toward peroxyl radicals [11,13]. As is the case for phenols, 3-pyridinols and 5-pyrimidinols can be substituted with electron-donating groups to weaken their O–H bonds and increase their rates of reaction with peroxyl radicals in a predictable fashion [11]. Based on these facts, we surmised that 3-pyridinols and 5-pyrimidinols would be ideal principal antioxidants in synergistic co-antioxidant systems with phenols. Herein we describe the rational design and kinetic characterization of such systems based on the combination of suitably substituted 3-pyridinols and 5-pyrimidinols (**4**–**9**) with conventional phenolic antioxidants (**10**–**12**).

## Results and Discussion

**Synthesis*.*** The preparation of compounds **4a**, **4b** and **5**–**8** involved installation of the aryl alcohol moiety as the final step via a Cu-catalyzed benzyloxylation/hydrogenolysis sequence on the corresponding pyri(mi)dyl halides, whereas the preparation of **4c**, **4d** and **9** followed a route starting from pyridoxamine, wherein the aryl alcohol is present throughout the sequence. Details are provided in the Experimental section and/or the cited references.

**Reactivity with peroxyl radicals*.*** To set up a rational framework for the design of co-antioxidant systems, the rate constants for the reactions of various pyridinols and pyrimidinols with peroxyl radicals (*k*_inh_) were measured by the well-established inhibited autoxidation of styrene (or cumene) in chlorobenzene at 303 K. These measurements also included experiments with three well-established phenolic antioxidants: 2,6-di-*tert*-butyl-4-methylphenol (BHT, **10**), 2,6-di-*tert*-butyl-4-methoxyphenol (DBHA, **11**), a hindered analogue of the widely employed BHA, and 2,2,5,7,8-pentamethylchroman-6-ol (PMHC, **12**), a synthetic analogue of α-tocopherol lacking its phytyl sidechain. While some of these rate constants have been reported in the literature, we felt it necessary to determine them all under the exact same conditions in order to be able to predict and/or rationalize observations made when the antioxidants are used in combinations. The results are given in Table 1. It must be pointed out that where previous data have been obtained under comparable conditions, our data are in excellent agreement – with *k*_inh_ values usually within a factor of two. As a result, the reactivity trends parallel those that have been observed before: the bicyclic naphthyridinol compounds **9a**–**c** generally possess the highest reactivities, followed by the aminopyridinols **4** and aminopyrimidinols **6**, and finally the alkoxypyridinols **5** and alkoxypyrimidinols **7**. A previously unstudied compound – the 2,4-dimethylpyrrole-substituted pyrimidinol **8** – was the least reactive pyri(mi)dinol we studied, with a rate constant almost 200 fold lower than that of the analogous dimethylamino-substituted pyrimidinol **6b** (*k*_inh_ = 4.4 × 10^4^ versus 7.4 × 10^6^ M^−1^s^−1^, respectively). Clearly, the 2,4-dimethylpyrrole substituent is not as electron-donating as a dimethylamino substituent (the O–H bond in **8** is 6.6 kcal/mol stronger than that in **6b**, vide infra). These results provide an explanation for the significant differences in the radical scavenging activities of pyridinols bearing these substituents in recently reported cell-based assays [18].

**Table 1:** Rate constants for the reactions of **4**–**12** with peroxyl radicals (*k*_inh_) at 303 K obtained from AIBN-initiated inhibited autoxidations of styrene (50% v/v) in either chlorobenzene (PhCl) or acetonitrile (CH_3_CN). O–H Bond dissociation enthalpies calculated using CBS-QB3 are given along with available experimental data where possible.

	*k*_inh_ (PhCl)		*k*_inh_ (CH_3_CN)		*k*_inh_ (PhCl)/*k*_inh_ (CH_3_CN)	BDE_OH_^calc^(^exp^)^a^ /kcal/mol
	/M^−1^s^−1^	*n*	/M^−1^s^−1^	*n*		

**4a**	(3.6 ± 0.6) × 10^6 b^	1.9	(5.4 ± 0.2) × 10^5^	2.0	7	77.9
**4b**	(1.4 ± 0.6) × 10^7 c^	1.9	(3.0 ± 0.3) × 10^6^	2.0	5	74.8 (75.9)
**4c**	(2.0 ± 1.0) × 10^6 d^	2.1	(3.1 ± 0.6) × 10^5^	2.0	6	78.0
**4d**	(8.5 ± 2.8) × 10^6 d^	1.9	(3.0 ± 0.4) × 10^6^	2.0	3	74.5
**5a**	(7.3 ± 0.4) × 10^4^	2.2^e^	(4.1 ± 0.3) × 10^4^	1.9^e^	18	82.4
**5b**	(4.4 ± 0.7) × 10^5 c^	2.1	(3.8 ± 0.9) × 10^4^	1.9^e^	12	78.9
**6a**	(2.0 ± 0.6) × 10^6 b^	2.1	(3.0 ± 0.7) × 10^5^	1.9	7	78.3
**6b**	(7.4 ± 0.6) × 10^6^	2.1	(1.0 ± 0.3) × 10^6^	1.8	7	75.6 (77.1)
**7a**	(3.1 ± 0.4) × 10^5^	2.0	(1.1 ± 0.6) × 10^4 f^	2.1^e^	28	80.9 (81.4)
**7b**	(3.7 ± 0.3) × 10^5^	2.0	(1.4 ± 0.5) × 10^4^	2.0^e^	26	80.9^g^
**8**	(4.4 ± 1.0) × 10^4^	2.0^e^	(1.3 ± 0.5) × 10^3^	n.d.	34	81.8
**9a**	(5.5 ± 3.1) × 10^7 h^	1.3	(9.2 ± 1.9) × 10^6^	1.7	6	74.9 (75.2^i^)
**9b**	(7.8 ± 0.8) × 10^7 h^	1.5	(1.3 ± 0.3) × 10^7^	1.7	6	75.0 (75.2^i^)
**9c**	(1.5 ± 0.2) × 10^7 j^	2.0	(2.9 ± 1.4) × 10^6^	2.0	5	75.4
**10**	(1.1 ± 0.2) × 10^4 k^	2.0^e^	n.d.	n.d.	-	78.7 (79.9^l^)
**11**	(1.1 ± 0.2) × 10^5 k^	2.0	(2.5 ± 1.0) × 10^4 f^	n.d.	4	75.5 (77.2^l^)
**12**	(3.2 ± 0.5) × 10^6 k^	2 ^l^	(6.5 ± 0.8) × 10^5 f^	2^m^	5	77.7 (77.1^l^)

^a^Experimental values (in benzene) obtained by REqEPR at 298 K are from [12–13] and have been corrected for the revised O–H BDE of phenol [19]. ^b^Values for **4a** and **6a** were previously determined as 4.8 × 10^6^ M^−1^s^−1^ and 1.1 × 10^6^ M^−1^s^−1^ at 303 K from the inhibited oxidation of styrene in PhCl and as 1.1 × 10^7^ M^−1^s^−1^ and 6.5 × 10^6^ M^−1^s^−1^ at 310 K in benzene by radical clock [16]. ^c^Values of 1.6 × 10^7^ M^−1^s^−1^ and 2.9 × 10^5^ M^−1^s^−1^ were previously reported for **4b** and **5b** from inhibited styrene oxidation in PhCl at 303 K [14]. ^d^Values for **4c** and **4d** of 3.3 × 10^6^ M^−1^s^−1^ and 8.7 × 10^6^ M^−1^s^−1^ measured by inhibited autoxidation of styrene in PhCl and of 1.6 × 10^6^ M^−1^s^−1^ and 1.4 × 10^7^ M^−1^s^−1^ in benzene at 310 K by radical clock [17]. ^e^Determined from the inhibited autoxidation of cumene at 303 K. ^f^Values of 7.9 × 10^2^ M^−1^s^−1^, 2.2 × 10^4^ M^−1^s^−1^ and 6.8 × 10^5^ M^−1^s^−1^were previously measured for **7a**, **11**, **12** from the autoxidation of styrene in acetonitrile at 303 K [20]. ^g^Assumed the same as **7a**. ^h^Values of 6.1 × 10^7^ M^−1^s^−1^ and 5.2 × 10^7^ M^−1^s^−1^ for **9a** and **9b** in benzene at 310 K were obtained by radical clock [15]. ^i^Measured for the analogue of **9a/b** with R = R’ = H. ^j^The value of 3.1 × 10^7^ M^−1^s^−1^ in benzene at 310 K was obtained by radical clock [15] for an analogue of **9c**. ^k^Values of 1.4 × 10^4^ M^−1^s^−1^, 1.1 × 10^5^ M^−1^s^−1^ and 3.8 × 10^6^ M^−1^s^−1^ were previously determined for **10**, **11** and **12** in the inhibited autoxidation of styrene in PhCl at 303 K [21]. ^l^From [22]. ^m^Used as reference value.

To provide further insight into the relative reactivities of these compounds we also carried out measurements of *k*_inh_ in acetonitrile as a representative polar solvent. We felt this was necessary since there is essentially no data available in the literature for the reactivity of the vast majority of these compounds in any media other than chlorobenzene (or benzene) and we wanted to examine the solvent-dependence of any synergism we observed (vide infra). The results demonstrate a significant kinetic solvent effect, which was most pronounced for the least reactive compounds. For example, *k*_inh_ for the methoxy-substituted pyridinol **5a** dropped by a factor of 18 on going from chlorobenzene to acetonitrile, while the reactivity of the more reactive *N*,*N*-dimethylamino-substituted pyridinol **4a** dropped only a factor of 7. Likewise, while *k*_inh_ for the 2,4-dimethyl-6-methoxy-3-pyridinol (**5b**) dropped 12-fold with the change in solvent, the reactivity of the equivalently-substituted, but less reactive, pyrimidinol **7a** dropped 28-fold.

**Scheme 4:**
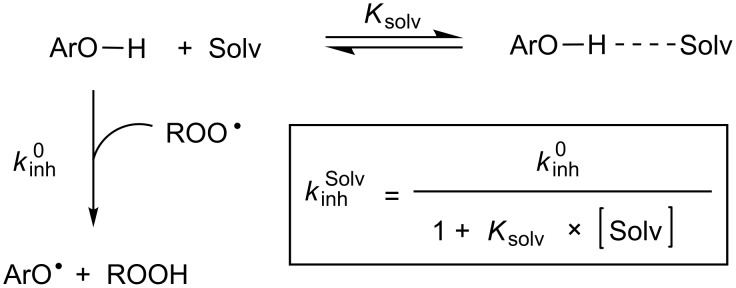
Model for kinetic solvent effects on the radical-trapping activity of phenolic antioxidants.

Ingold has clearly demonstrated that formal H-atom transfer reactions of the type X–H + Y^•^ → X^•^ + H–Y, where X is an electronegative atom, can experience a large kinetic solvent effect (KSE). In fact, these reactions are slowed down in hydrogen-bond accepting (HBA) solvents as a result of H-bond formation between X–H and the solvent since the H-bonded complex is essentially unreactive to the abstracting radical; hence only the “free” fraction of X–H in solution can react [23–26]. This KSE (illustrated in Scheme 4) is known to have major impact on the performance of phenolic antioxidants [2,10,27–28].

Since the formation of the H-bonded complex is driven by both the HBA ability of the solvent and the hydrogen-bond donating (HBD) ability of the H-atom donor, the KSEs evident in the data above reflect the H-bond acidity of the various radical-trapping antioxidants we have studied. On quantitative grounds, an empirical relationship between *K*_solv_ and the HBD ability of the compound is provided by Abraham's equation (Equation 1), where 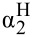 and 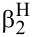 are empirical solvatochromic parameters (range: 0 to 1) quantifying the HBD and HBA ability of the two interacting partners (e.g. the phenol and the solvent), respectively, in the formation of a 1:1 H-bonded complex [29–30].

[1]
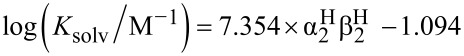


An 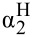 value of 0.37 [24] has been reported for α-tocopherol (expectedly identical to PMHC, **12**), while they have been estimated as 0.50 and 0.55 for compounds **4a** and **6a** respectively [2,10], consistent with the larger KSEs on the reactions of the latter (ca. 7) relative to the former (ca. 5).

**Figure 2:**
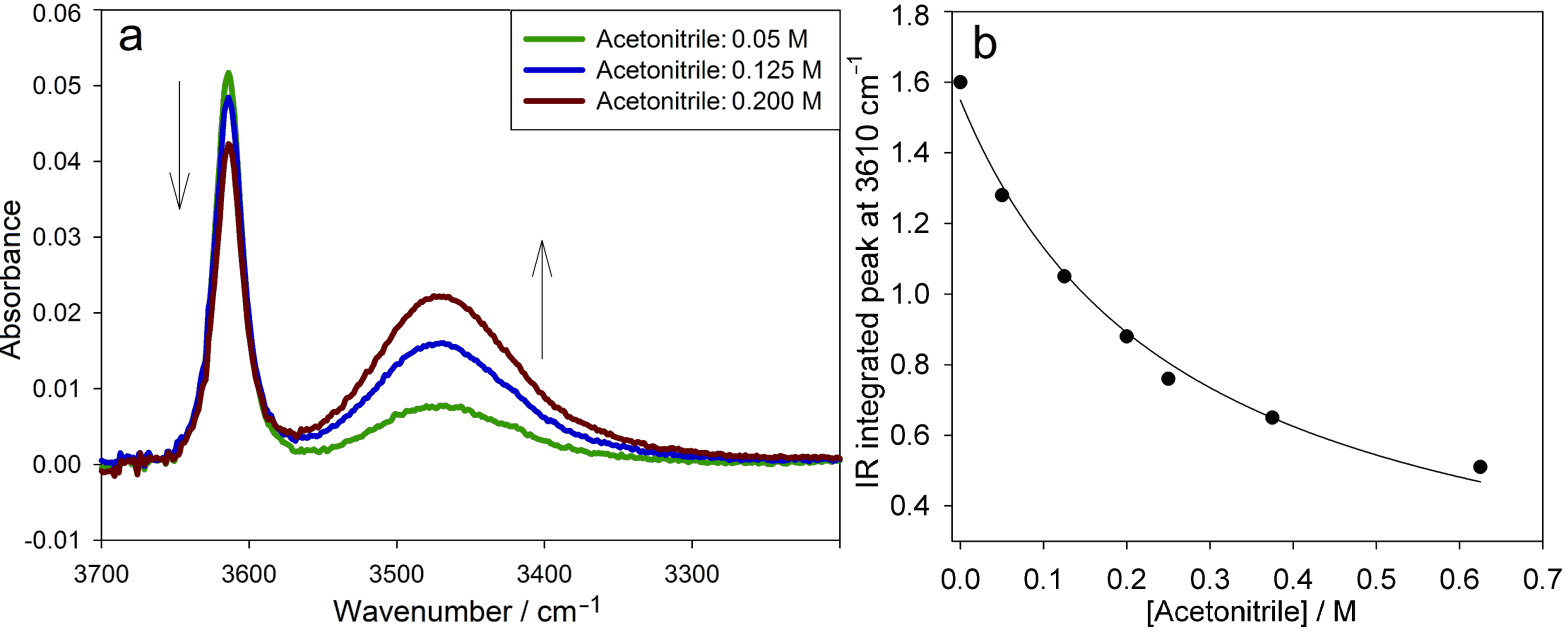
The O–H stretching region of representative FTIR spectra of compound **6b** (10 mM) in CCl_4_ containing increasing amounts of acetonitrile as co-solvent (a) and corresponding plot of the integrated signal at 3610 cm^−1^ versus the concentration of acetonitrile, fit to Equation 2 (b).

**FTIR measurements.** To put the HBD ability of the pyridinols and pyrimidinols on solid quantitative ground, we performed independent (non-kinetic) measurements of *K*_solv_ for three representative compounds (**5b**, **6b** and **7b**) in three reference solvents of different HBA ability [30]: acetonitrile (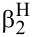 = 0.44), ethyl acetate (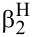 = 0.45), and dimethyl sulfoxide (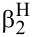 = 0.78) using IR spectroscopy [25]. Representative results are shown in Figure 2.

Addition of a HBA solvent to solutions of the pyri(mi)dinols in non-H-bonding CCl_4_ resulted in the progressive decrease of the IR signal corresponding to the “free” O–H stretch (~3610 cm^−1^), accompanied by the growth of a broad intense band at lower frequency attributed to the O–H stretch of the H-bonded species (Figure 2a). By fitting the data corresponding to the integrated IR signal for the free O–H versus the concentration of the HBA co-solvent to the expression in Equation 2 as illustrated in Figure 2b, the values of *K*_solv_ collected in Table 2 could be obtained.

[2]
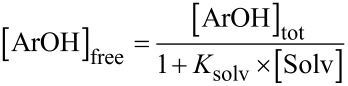


It should be noted that, for any of the compounds that were investigated, there is good agreement between the 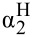 values obtained by Equation 1 from equilibrium constants in ethyl acetate and DMSO, while the value measured for acetonitrile is consistently lower. Indeed, for each of these compounds, *K*_solv_ measured for acetonitrile is lower than that for ethyl acetate despite the fact that the two solvents are attributed essentially the same HBA ability by Abraham’s β_2_^H^ scale (0.44 versus 0.45). A similar trend is observed in available literature kinetic data; the rate constants for formal H-atom transfer from a variety of phenols to a variety of radicals (e.g*.* alkyl, alkoxyl, peroxyl and DPPH) is consistently higher in acetonitrile than in ethyl acetate [23–28] strongly suggesting that the 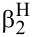 value for acetonitrile needs revision. As a result, we suggest averaging the 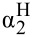 values determined for **5b**, **6b** and **7b** in EtOAc and DMSO, resulting in 0.55, 0.53 and 0.65, respectively. Such values are in line with other phenol-type antioxidants [2,10]. The same values can then be used as inputs in Equation 1 to obtain an average 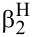 of 0.39 for acetonitrile. This value is in accord with that obtained from kinetic measurements: for instance, using the reaction of α-tocopherol with *tert*-butoxyl radicals as model, it was shown that acetonitrile has the same HBA ability as water [31], which is attributed a reliable 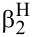 = 0.38 [29–30]. As such, we recommend a value of 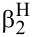 of 0.39 for acetonitrile.

**Table 2:** FTIR measured equilibrium constants at 298 K for H-bonding of solvents with selected antioxidants (*K*_solv_) and corresponding 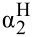 values calculated by Equation 1.

	Solvent	*K*_solv_/M^−1^	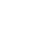	KSE^a^

**5b**	CH_3_CN	3.1 ± 0.2	0.49	
	EtOAc	5.5 ± 0.3	0.55	
	DMSO	116.1 ± 11.2	0.55	
		average^b^	0.55	12
**6b**	CH_3_CN	3.0 ± 0.3	0.49	
	EtOAc	4.7 ± 0.5	0.53	
	DMSO	95.0 ± 5.9	0.53	
		average^b^	0.53	7
**7b**	CH_3_CN	6.9 ± 1.8	0.60	
	EtOAc	14.1 ± 0.9	0.68	
	DMSO	285.0 ± 9.6	0.62	
		average^2^	0.65	26

^a^KSE = kinetic solvent effect, taken from data in Table 1. ^b^Average of the data in EtOAc and DMSO only, see text.

**Computational thermodynamics*.*** The rational design of synergistic co-antioxidant mixtures requires knowledge of not only the kinetics of the reactions of the antioxidants with peroxyl radicals, but also the relative stabilities of the antioxidant-derived radicals, since synergism relies on the position of the equilibrium of reaction 6 (Scheme 3) [8–9], which is related to the difference in the O–H BDEs of the equilibrating antioxidants as in Equation 3.

[3]
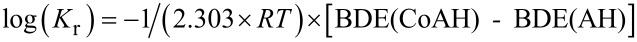


In order to complete the necessary framework of kinetic and thermodynamic data, we computed the O–H BDEs of compounds **4**–**12** using quantum chemical methods. The calculations were carried out at the CBS-QB3 level of theory [32], since this approach has been shown to provide highly accurate O–H BDEs in phenols and related compounds [19,33–35]. The results of these calculations are given in Table 1 alongside the limited experimental data obtained using the radical equilibration EPR (REqEPR) technique [12–13,22]. The calculated BDEs are in very good agreement with the experimental values, with systematic deviations between 0.2 and 1.7 kcal/mol and, most importantly, they allow insight into the position of equilibrium (reaction 6, Scheme 3) unfettered by differing experimental conditions.

**Co-antioxidant systems*.*** Given the foregoing kinetic and thermodynamic data, we next set out to design and test representative co-antioxidant mixtures. For simplicity we investigated only binary AH/co-AH mixtures. The baseline strategy consisted of selecting a pyridinol or a pyrimidinol as principal antioxidant (AH) capable of providing maximum radical-trapping kinetics to the mixture (higher *k*_inh_), but at the same time a sufficiently high O–H BDE to be regenerated by the co-antioxidant, co-AH (vide supra), which was selected among the conventional phenols **10**–**12**.

**Figure 3:**
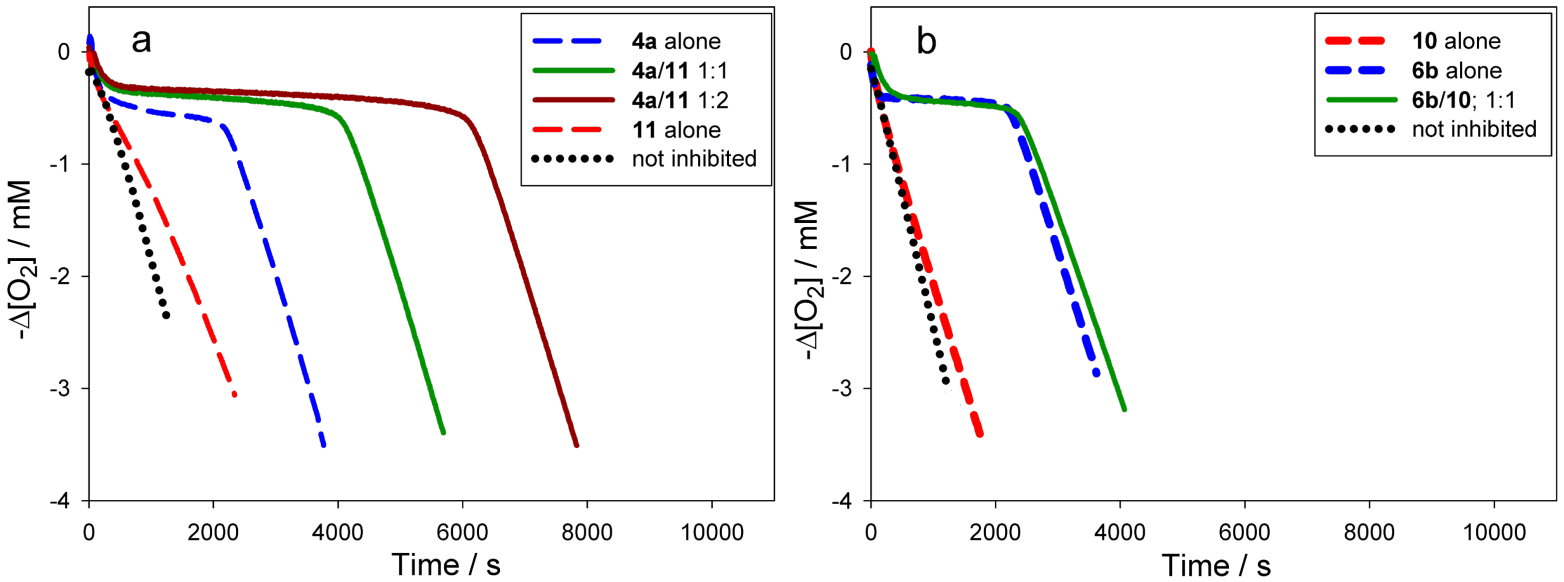
Oxygen-uptake plots recorded during the AIBN initiated autoxidation of styrene in chlorobenzene (50% v/v) at 303 K in the absence or presence of: (a) **4a** (6.2 × 10^−6^ M), **11** (6.2 × 10^−6^ M) or a mixture of **4a** (6.2 × 10^−6^ M) with either one or two equivalents of **11**; (b) **6b** (6.2 × 10^−6^ M), **10** (6.2 × 10^−6^ M) or a mixture of **6b** (6.2 × 10^−6^ M) and one equivalent of **10**.

The autoxidation of an organic substrate (e.g. styrene) thermally initiated at a constant rate, *R*_i_, by an azo-initiator will consume oxygen at a constant rate in the absence of an inhibitor. In the presence of a very effective antioxidant such as **4a** (*k*_inh_ = 3.6 × 10^6^ M^−1^s^−1^, see Table 1) a plot of oxygen uptake versus time shows a clear inhibition period of length τ_0_ that depends on the concentration of AH and the stoichiometric factor (*n* ~ 2 for all tested antioxidants, see Table 1). During the inhibited period (cf. Figure 3), i.e. until AH has been consumed, the rate of oxygen consumption is almost completely suppressed, after which it resumes at the uninhibited rate. Figure 3 also shows that an equivalent amount of a modest antioxidant such as **11** (*k*_inh_ = 1.1 × 10^5^ M^−1^s^−1^, see Table 1) does not produce a neat inhibition of the autoxidation under the same conditions, but instead simply retards oxygen uptake, since the propagation of autoxidation can compete effectively with inhibition. However, when equimolar amounts of **4a** and **11** are present, a clear inhibited period is observed – as was the case for **4a** alone, but its duration is twice what it was in the absence of the equivalent of **11**. This result implies that **11** can regenerate **4a** from its corresponding aryloxyl radical. This reaction is driven by the fact that **4a** has an O–H bond which is 2.4 kcal/mol stronger (77.9 kcal/mol) than the O–H bond in **11** (75.5 kcal/mol). The addition of another equivalent of **11** extends the inhibited period to three times that of **4a** alone, clearly demonstrating that it is effectively used as the sacrificial reductant during the inhibited period.

The duration of the inhibited period (τ) is related to the concentration ratio of the principal antioxidant and co-antioxidant by a proportionality constant α (Equation 4), which represents the efficiency with which AH is regenerated by co-AH, which can be written in terms of the rate constants of the relevant competing reactions in Scheme 3 as in Equation 5; thus, its value may lie between 0 (no regeneration of AH by co-AH) and 1 (complete regeneration of AH by co-AH).

[4]
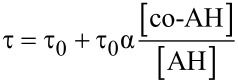


[5]
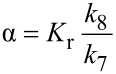


In other words, α is a measure of synergism in the co-antioxidant system. Since the ratio *k*_8_/*k*_7_ (see Scheme 3) for phenolic antioxidants is normally ~1 [8], the efficiency of regeneration depends almost entirely on *K*_r_. Moreover, since H-atom transfer between phenols usually proceeds with a negligible change in entropy, α depends largely on the difference in the O–H BDEs of the two co-antioxidants (Equation 3). If *K*_r_ << 1, regeneration will be inefficient and no synergism will be observed; under those circumstances the co-antioxidants will simply behave in an additive fashion, as illustrated in Figure 3b for the combination of **6b** (BDE = 75.6 kcal/mol) and **10** (BDE = 78.7 kcal/mol).

**Figure 4:**
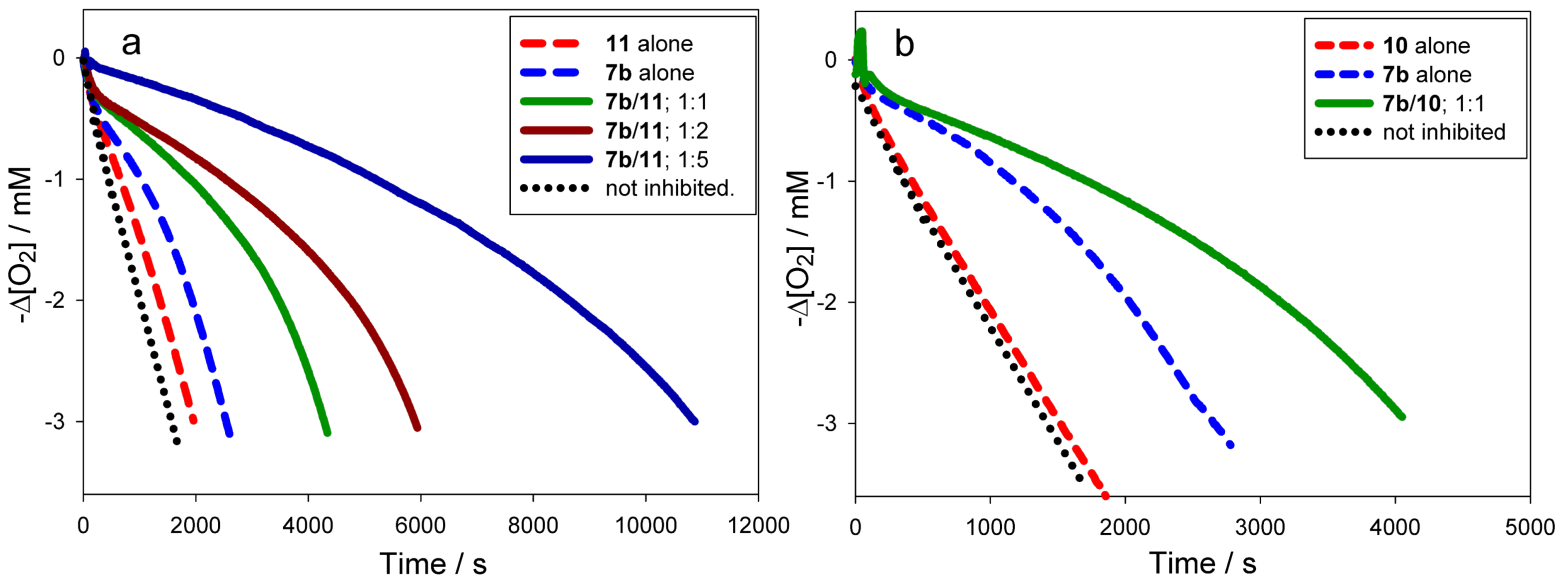
Oxygen-uptake plots recorded during the AIBN initiated autoxidation of styrene in chlorobenzene (50% v/v) at 303 K in the presence of compound **7b** and/or **11** (either 6.1 × 10^−6^ M) (a) and corresponding plots using compound **10** as co-antioxidant (b).

Although, in principle, synergism can occur with any AH/co-AH ratio (e.g. Figure 4a), the efficiency often changes to some extent as a function of such ratio, as well as with the actual experimental conditions. For simplicity, all co-antioxidant mixtures were investigated under comparable settings in the low μM range with AH/co-AH ratios of 1:1 and 1:2. As can be seen from Table 3, several of the antioxidant mixtures investigated showed good synergism in chlorobenzene (α > 0.5); particularly the couples **4a**/**11**, **6a**/**11**, **6a**/**12**, **6b**/**11**, **6b**/**12**, **7a**/**11**, **7a**/**10** (similar to **7b**/**11**, **7b**/**10**), **9c**/**12**. From our results we can conclude that, in general, ΔBDE needs to be > −1 kcal/mol to expect synergism based on the equilibrium of reaction 6. Not surprisingly, regeneration was more efficient when a pyrimidinol was used as the principal antioxidant due to the higher O–H BDEs of the pyrimidinols relative to equivalently substituted pyridinols. On the other hand, it should be noted that the efficiency α is not the only relevant parameter in determining the overall efficacy of a co-antioxidant mixture, since the apparent *k*_inh_ of the mixture will be identical to that of the most reactive antioxidant in the mixture [8]. For instance, the mixture **4a**/**11** (Figure 3a) is a significantly better antioxidant system than mixtures of **7b**/**11** (Figure 4a) and **7b**/**10** (Figure 4b), despite all systems having α = 1.

**Table 3:** Regeneration efficiency (α) of a principal antioxidant (AH) by a co-antioxidant (co-AH) in the inhibited autoxidation of styrene in chlorobenzene or acetonitrile (50% v/v) at 303 K.^a^

AH	co-AH	ΔBDE (AH-CoAH)	α (PhCl)	α (CH_3_CN)

**4a**	**11**	+2.4	1.0 ± 0.1	0.5 ± 0.1
**4c**	**11**	+2.5	~0.1	~0
**4d**	**11**	−1.0	~0.1	~0.1
	**10**	−4.2	~0	~0
**6a**	**11**	+2.8	1.0 ± 0.1	0.8 ± 0.1
	**12**	+0.6	0.8 ± 0.2	0.4 ± 0.2
**6b**	**10**	−0.4	0.5 ± 0.1	~0
	**11**	+0.1	0.7 ± 0.1	0.5 ± 0.1
	**10**	−2.1	~0	~0
**7a**	**11**	+4.4	0.9 ± 0.1	n.d.^b^
	**10**	+2.2	0.9 ± 0.1	0.3 ± 0.1
**7b**	**11**	+4.4	1.0 ± 0.1	0.7 ± 0.2
	**10**	+2.2	1.0 ± 0.1	n.d*.*^b^
**9c**	**12**	−2.3	0.6 ± 0.2	0.5 ± 0.1

^a^Values are averaged on at least three independent experiments with AH/co-AH ratios of 1:1 and 1:2, in the concentration range 2–10 μM both for AH and co-AH. ^b^n.d*.* = not determined.

**Figure 5:**
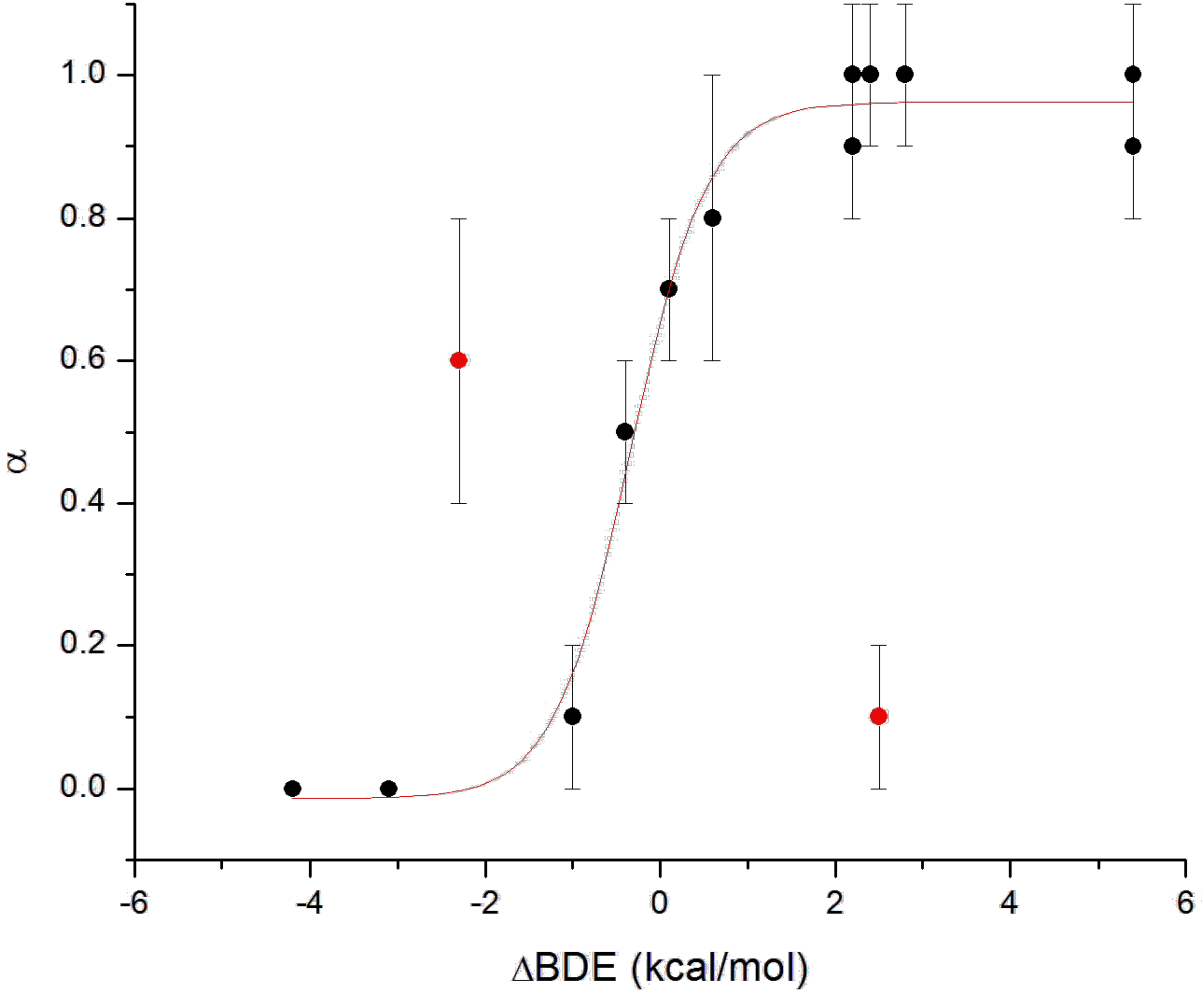
Regeneration efficiencies (α) observed in autoxidations of styrene in chlorobenzene (50% v/v) at 303 K inhibited by co-antioxidant mixtures as a function of the difference in the O–H BDEs of the principal (AH) and co-antioxidant (Co-AH) [ΔBDE = BDE(AH) – BDE(Co-AH)]. The data highlighted in red correspond to the combinations of **9c**/**12** and **4c**/**11**.

Since synergistic activity requires a favourable ΔBDE (vide supra), it seems reasonable to expect that the values of α should correlate with ΔBDE. Such a correlation is shown in Figure 5, in which there appears to be a clear sigmoidal relationship between α and ΔBDE. The correlation is sigmoidal since below ΔBDE values of ca. −1 kcal/mol little to no regeneration is observed, whereas above ca. 1 kcal/mol, regeneration is essentially quantitative.

There are two data points that do not lie on the correlation: the combinations of **4c** with **11**, and **9c** with **12**. The latter has ΔBDE = −2.3 kcal/mol, implying that **9c** should not be regenerated by **12**. However, because the phenolic co-antioxidant used in this case (**12**) is a very reactive antioxidant itself (*k*_inh_ = 3.2 × 10^6^ M^−1^s^−1^ compared to 1.5 × 10^7^ M^−1^s^−1^ for **9c**) it also gives a pronounced inhibited period under the reaction condition (see Supporting Information File 1 for oxygen uptake plots). As such, the additive contributions of **9c** and **12** give the appearance of synergism where only additivity exists. It should also be pointed out that although the simple additive contributions of the highly reactive antioxidants should give α = 1, a value of only ca. 0.6 is observed. This is likely due to the consumption of some **12** by autoxidation at higher concentrations of **9c** due to the longer inhibition period, which is known to lead to lower stoichiometric factors for highly similar compounds (i.e. **9a**, **9b**) [17]. This leaves the **4c**/**11** data point as the only real outlier; since it has ΔBDE = +2.5 kcal/mol, the much more reactive **4c** should be regenerated by the less reactive **11**. This result is puzzling in light of the fact that the structurally similar pyridinol **4a**, which has an essentially identical O–H BDE, is fully regenerated by **11**. The only structural feature which distinguishes **4c** from the other pyridinols and pyrimidinols in Table 3 (and Figure 5) that have positive ΔBDE values (and therefore α ~ 1) is in the conformation of the substituent at the 4-position relative to the reactive hydroxyl moiety. Due to steric interactions between the adjacent ring methyl, the dimethylamino substituent in **4c** is rotated out of the plane of the aromatic ring [17]. On the basis of theoretical calculations, H-atom transfer between phenol and the phenoxyl radical is believed to be a proton-coupled electron transfer reaction, occurring via an approximately planar transition state wherein the unpaired electron is delocalized across both phenyl rings [36]. As such, it is difficult to envision how the conformation of the dimethylamino substituent in **4c** may slow the analogous reaction between the radical derived from **4c** and **11** relative to the other reaction couples. However, it should be pointed out that theoretical calculations on the phenol/phenoxyl H-atom transfer reaction do not include substituents in the ortho positions relative to the phenolic oxygen, which may change the transition state structure substantially.

Regardless of whether the foregoing rationalization is in fact correct, the lower than expected regeneration efficiency observed for the combination of **4c** and **11** underscores the fact that the actual value of α depends not only on the equilibrium in reaction 6 (Scheme 3) – and hence the ΔBDE – but also on the absolute rates of the other reactions depicted in Scheme 3 [8]. In this connection, it is important to note that the values of α we measured were generally lower in acetonitrile than in chlorobenzene despite the fact that ΔBDE is expected to increase on going from chlorobenzene to acetonitrile due to the stronger H-bonding of the pyridinols and pyrimidinols (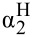 ~ 0.5–0.7) to acetonitrile (vide supra) as compared to the sterically hindered phenols such as **10** and **11** (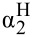 ~ 0.2) [37]. The drop in α can only be explained by considering Scheme 3 in more detail. For regeneration of the principal antioxidant AH (the pyridinol/pyrimidinol) to occur, it is necessary that equilibrium of reaction 6 is faster than reaction 7 (Scheme 3), i.e. *k*_7_ × [ROO^•^]_SS_ < *k*_r_ × [co-AH]. In the presence of a good antioxidant AH (rapidly trapping peroxyl radicals by reaction 4 (Scheme 3)) this condition is easily met [8]. However, if the reactivity of AH is hampered by H-bonding to the solvent, the steady state concentration of peroxyl radicals may grow sufficiently to react competitively with co-AH, thereby decreasing the efficiency of regeneration [38].

## Conclusion

Herein we have provided the kinetic and thermodynamic rationale for the design of synergistic co-antioxidant systems employing highly reactive 3-pyridinol or 5-pyrimidinol antioxidants in combination with less reactive, but much less expensive, phenolic antioxidants. In several cases, the approach has shown to equal the performance of the best co-antioxidant systems designed by nature, such as the tocopherol/ascorbate system [5] or the tocopherol/catechol system [8]. In general, the most effective individual antioxidants, e.g. the bicyclic pyridinols (**9a–c**), pyridinols (**4b**) and pyrimidinols (**6d**) are not good partners for co-antioxidant systems because their O–H BDEs (74.8–75.6 kcal/mol) are too low. Instead, the slightly less reactive pyridinols and pyrimidinols (e.g. **4a**, **6a**, **7a**/**7b**), which have much stronger O–H bonds (>78 kcal/mol), are the ideal candidates to be used with abundant, persistent phenols such as BHT (**11**). We anticipate that this work will prompt the use of antioxidant mixtures based on 3-pyridinol and 5-pyrimidinol antioxidants, in order to take advantage of the greater reactivities of these compounds, but to minimize the cost of doing so by making use of the inexpensive phenolic antioxidants typically used in industrial/commercial applications to regenerate them in situ.

## Experimental

**Materials*.*** Solvents were of the highest grade commercially available (Fluka/Aldrich) and were used as received. 2,2,5,7,8-Pentamethyl-6-chromanol (PMHC, **12**, 97%) was commercially available (Aldrich) and used without further purification. Commercial 2,6-di-*tert*-butyl-4-methylphenol (BHT, **10**, 98%) and 2,6-di-*tert*-butyl-4-methoxyphenol (DBHA, **11**, 97%) were re-crystallized from hexane. Commercially available 2,2'-azodiisobutyronitrile (AIBN ≥98%) was recrystallized from hexane and stored at −20 °C. Cumene (98%) and styrene (≥99%) were distilled under reduced pressure and percolated twice through silica and alumina prior to use. All solutions were prepared fresh immediately prior to use.

**Synthesis*.*** Compounds **4a**, **4b**, **6a** and **6b** were prepared as described in [39]. Compounds **4c** and **4d** were prepared as described in [17]. Compound **5b** was prepared as in [14]. Compound **7a** was prepared as in [13]. Compounds **9a** and **9b** were prepared as in [15], whereas compound **9c** was prepared as in [40].

**3-Hydroxy-6-methoxypyridine (5a).** A solution of 3-benzyloxy-6-methoxypyridine [39] in MeOH was treated with 10% Pd/C and the resulting black suspension was stirred at room temperature under an atmosphere of H_2_ (1 atm) overnight. The catalyst was removed by filtration through a pad of celite and the filtrate was concentrated under reduced pressure. The crude residue obtained was subjected to flash chromatography on silica gel (eluent: ethyl acetate/hexanes) and the product isolated in quantitative yield. ^1^H NMR (CDCl_3_) δ 9.32 (br s, 1H, exchanges with D_2_O), 7.75 (s, 1H), 7.25 (d, *J* = 8.6 Hz, 1H), 6.56 (d, 8.6 Hz, 1H), 3.84 (s, 3H); ^13^C NMR (CDCl_3_) δ 54.2, 111.0, 128.7, 132.3, 148.2, 158.3; HRMS (EI^+^) *m*/*z*: calcd for C_6_H_7_NO_2_, 125.0477; found, 125.0484.

**5-Hydroxy-2-octyloxy-4,6-dimethylpyrimidine (7b). ***O*-Octylisouronium trifluoromethanesulfonate (5.1 g, 15.8 mmol) was dissolved in dry DMF (30 mL), and 3-acetoxy-2,4-pentanedione (2.5 g, 15.8 mmol) was added along with sodium acetate (1.14 g, 15.8 mmol), and the mixture stirred for 24 hours at 70 °C. Water (200 mL) was then added, the pH adjusted to ~5, and the organics extracted with EtOAc (3 × 100 mL). The organic layers were combined, dried over MgSO_4_ and concentrated under reduced pressure. The product was then recrystallized from CH_3_CN to yield 35% **7b**. ^1^H NMR (CDCl_3_) δ 4.15 (t, *J* = 6.6 Hz, 2H), 2.32 (s, 6H), 1.63 (m, 2H), 1.32 (br m, 2H), 1.17 (m, 8H), 0.78 (t, 6.5 Hz); ^13^C NMR (CDCl_3_) δ 14.1, 18.8, 22.6, 26.0, 29.0, 29.2, 29.3, 31.8, 67.4, 142.5, 156.4, 158.3; HRMS (EI^+^) *m*/*z*: calcd for C_14_H_24_N_2_O_2_, 252.1838; found, 252.1836.

**5-Hydroxy-2-(2,5-dimethyl-1*H*-pyrrol-1-yl)-4,6-dimethylpyrimidine (8).** A solution of 5-benzyloxy-2-(2,5-dimethyl-1*H*-pyrrol-1-yl)-4,6-dimethylpyrimidine [41] in MeOH was treated with 10% Pd/C and the resulting black suspension was stirred at room temperature under an atmosphere of H_2_ (1 atm) overnight. The catalyst was removed by filtration through a pad of celite and the filtrate was concentrated under reduced pressure. The crude residue obtained was subjected to flash chromatography on silica gel (eluent: ethyl acetate/hexanes) and the product isolated in quantitative yield. ^1^H NMR (CDCl_3_) δ 2.15 (s, 6H), 2.43 (s, 6H), 5.71 (s, 2H); ^13^C NMR (CDCl_3_) δ 13.2, 18.4, 107.4, 128.6, 145.7, 149.3, 155.3; HRMS (EI^+^) *m*/*z*: calcd for C_12_H_15_N_3_O, 217.1215; found, 217.1217.

**Autoxidation studies*.*** The chain-breaking antioxidant activity of the title compounds was evaluated by monitoring the course of thermally initiated inhibited autoxidations of either styrene or cumene (RH) in chlorobenzene or acetonitrile. The autoxidation experiments were performed in a oxygen-uptake apparatus already described elsewhere [42–44]. In a typical experiment, an air-saturated mixture of styrene or cumene in acetonitrile or chlorobenzene (50% v/v) containing AIBN (1–5 × 10^−2^ M) was equilibrated with the reference solution containing also an excess of PMHC (1 × 10^−2^ M) in the same solvent at 30 °C. After equilibration, a concentrated solution of the antioxidant (final concentration 1–10 × 10^−6^ M) was injected into both the sample flasks, and the oxygen consumption of the sample was measured. From the rate of oxygen consumption during the inhibited period (*R*_inh_), *k*_inh_ values were obtained by using Equation 6 [44], where *R*_0_ is the rate of oxygen consumption in the absence of antioxidants, *R*_i_ is the initiation rate (in the range 2–10 × 10^−9^ Ms^−1^), 2*k*_t_ is the bimolecular termination rate constant of styrylperoxyl or cumylperoxyl radicals (4.2 × 10^7^ and 4.6 ×10^4^ M^−1^s^−1^ respectively) [21,43] and *n* is the stoichiometric coefficient of the antioxidant. The *n* coefficient was determined experimentally from the length of the inhibited period (τ) by Equation 7.

[6]
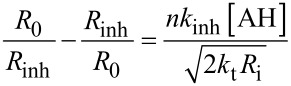


[7]
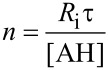


A similar procedure was employed to investigate the kinetics of the antioxidant mixtures. The efficiency, α, was determined from the oxygen uptake plots by the extension of the inhibition period according to Equation 4. In cases where no clear inhibition period was observed, α was obtained by fitting the experimental traces with numerical simulations based on Scheme 3 using Gepasi 3.0 software, as previously described [45].

**FTIR spectroscopy*.*** Spectra were recorded at 298 K in a Nicolet Protegé 460 FTIR spectrometer under nitrogen atmosphere using a sealed KBr cell with optical path of 0.5 mm. Solutions of the test compound (10 mM) in CCl_4_ and in CCl_4_/HBA-solvent mixtures were analyzed in absorbance mode and the blank spectrum of the corresponding solvent mixture was subtracted. The signal in the “free” O–H stretching region at ca. 3610 cm^−1^ was manually integrated after manual baseline correction and plotted versus the concentration of the HBA solvent and fit to Equation 2 [25]. In the case of compound **6b**, similar analysis was repeated using IR peak height in place of peak area and essentially indistinguishable results were obtained. In order to confirm the absence of self-association of the test compounds and to calibrate the spectrometer response, linear regression plots (Absorbance versus [ArOH]) in CCl_4_ were preliminarily recorded in the range 1–10 mM. Deviation from linearity was observed only in the case of **7b**, allowing the determination of its self-association equilibrium constant as *K*_self_ = 121 ± 10 M^−1^. Therefore, its H-bonding to the solvent was analyzed as described above using Equation 8 (see Supporting Information File 1 for further details).

[8]
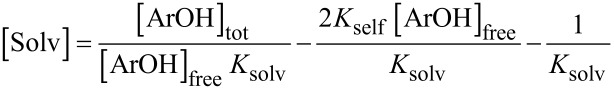


## Supporting Information

File 1 Additional experimental details, oxygen-uptake plots and FTIR spectra, as well as cartesian coordinates for calculated structures.
