# Supplementary material for: 3-Pyridinols and 5-pyrimidinols: Tailor-made for use in synergistic radical-trapping co-antioxidant systems
Source: Beilstein J Org Chem. 2013 Dec 4;9:2781–92. doi: 10.3762/bjoc.9.313 (PMC3869267; doi:10.3762/bjoc.9.313)
Supplement: File 1 — Additional experimental details, oxygen-uptake plots and FTIR spectra, as well as cartesian coordinates for calculated structures. [file Beilstein_J_Org_Chem-09-2781-s001.pdf]

Supporting Information

for

3-Pyridinols and 5-pyrimidinols: Tailor-made for use in synergistic radical-trapping co-antioxidant systems

Luca Valgimigli[1,*], Daniele Bartolomei[1], Riccardo Amorati[1], Evan Haidasz[2], Jason J. Hanthorn[2], Susheel J. Nara[2], Johan Brinkhorst[2], and Derek A. Pratt[2,*]

Address: [1]Department of Chemistry "G. Ciamician", University of Bologna, Via S. Giacomo 11, I-40126 Bologna, Italy and [2]Department of Chemistry, University of Ottawa, 10 Marie Curie Pvt., Ottawa, Ontario, Canada K1N 6N5

Email: Luca Valgimigli* - luca.valgimigli@unibo.it; Derek A. Pratt* - dpratt@uottawa.ca

* Corresponding author

Additional experimental details, oxygen-uptake plots and FTIR spectra, as well as cartesian coordinates for calculated structures

Contents

Oxygen consumption plots for inhibited autoxidations S2

FTIR spectra and fittings S6

Examples of kinetic modelling of autoxidations S9

Computational data S10

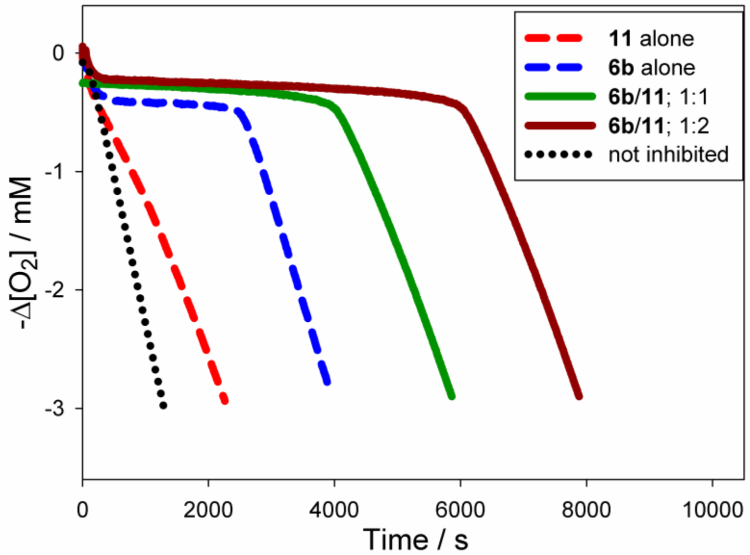

Figure S1: Oxygen-uptake plots recorded during the AIBN initiated autoxidation of styrene in chlorobenzene (50% v/v) at 303 K in the absence or presence of compound **6b** or **11** (either 6.2 × 10^{-6} M), or of a mixture of **6b** (6.2 × 10^{-6} M) and one or two equivalents of **11**.

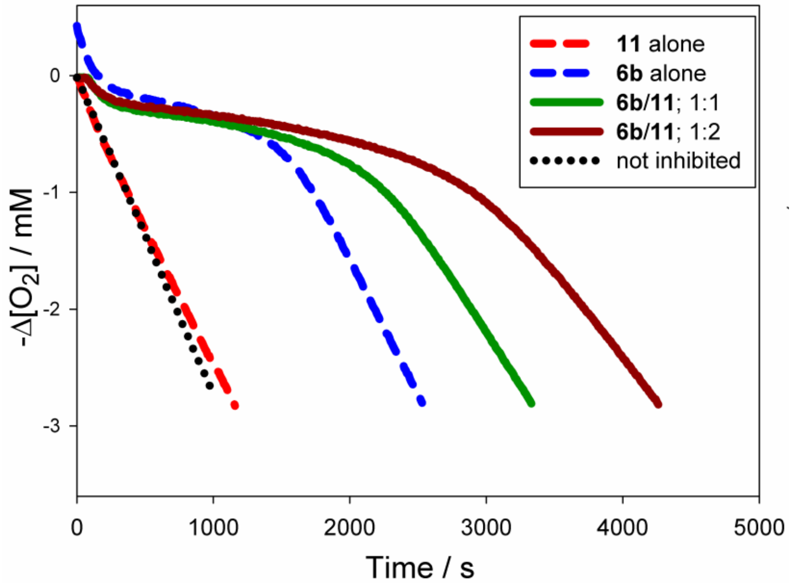

Figure S2: Oxygen-uptake plots recorded during the AIBN initiated autoxidation of styrene in acetonitrile (50% v/v) at 303 K in the absence or presence of compound **6b** or **11** (either 6.2 × 10^{-6} M), or of a mixture of **6b** (6.2 × 10^{-6} M) and one or two equivalents of **11**.

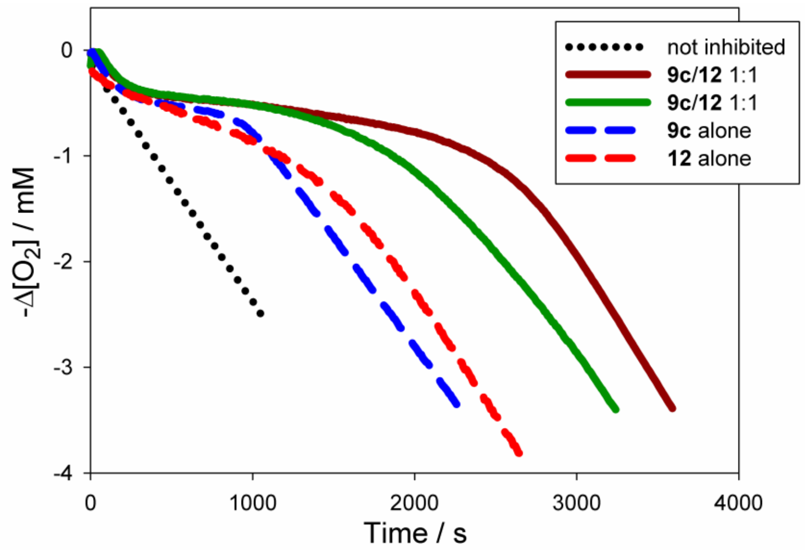

Figure S3: Oxygen-uptake plots recorded during the AIBN initiated autoxidation of styrene in acetonitrile (50% v/v) at 303 K in the absence or presence of compound **9c** or **12** (either 2.5 × 10^{-6} M), or of a mixture of **9c** (2.5 × 10^{-6} M) and one or two equivalents of **12**.

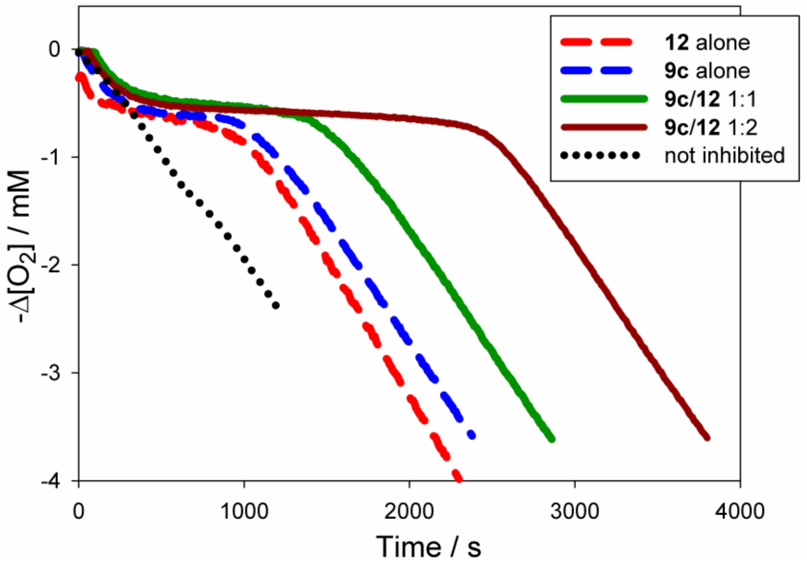

Figure S4: Oxygen-uptake plots recorded during the AIBN initiated autoxidation of styrene in chlorobenzene (50% v/v) at 303 K in the absence or presence of compound **9c** or **12** (either 2.5 × 10^{-6} M), or of a mixture of **9c** (2.5 × 10^{-6} M) and one or two equivalents of **12**.

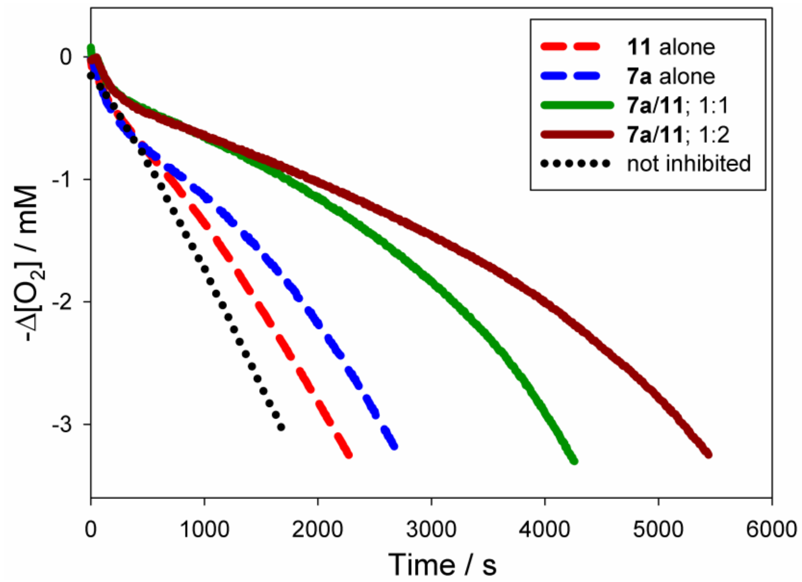

Figure S5: Oxygen-uptake plots recorded during the AIBN initiated autoxidation of styrene in chlorobenzene (50% v/v) at 303 K in the absence or presence of compound **7a** or **11** (either 6.2×10^{-6} M), or of a mixture of **7a** (6.2×10^{-6} M) and one or two equivalents of **11**.

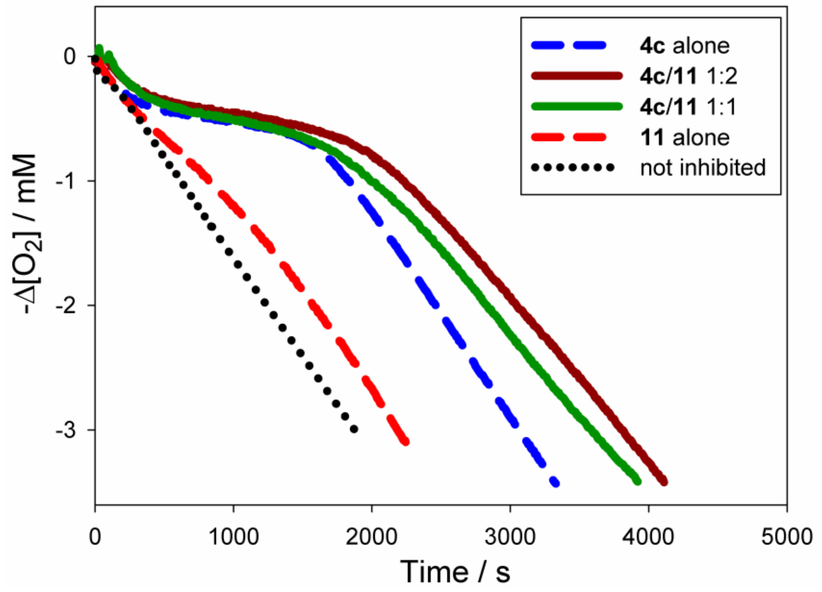

Figure S6: Oxygen-uptake plots recorded during the AIBN initiated autoxidation of styrene in chlorobenzene (50% v/v) at 303 K in the absence or presence of compound **4c** or **11** (either 4.9×10^{-6} M), or of a mixture of **4c** (4.9×10^{-6} M) and one or two equivalents of **11**.

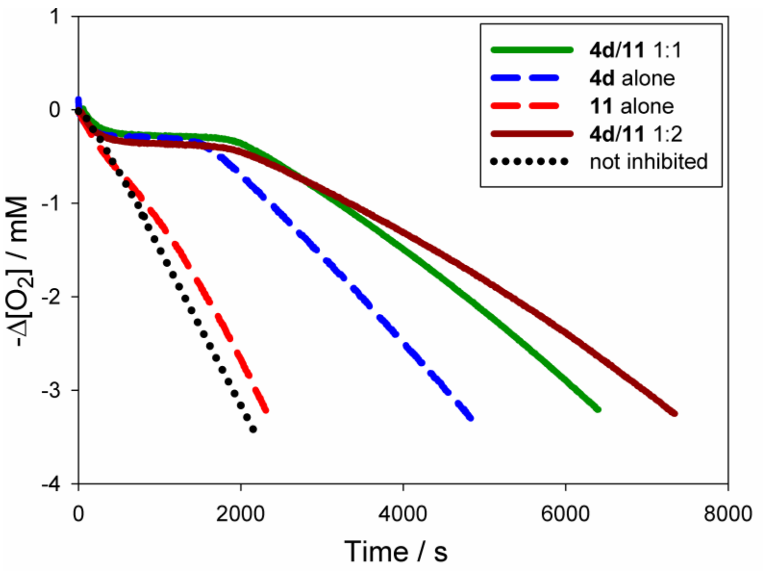

Figure S7: Oxygen-uptake plots recorded during the AIBN initiated autoxidation of styrene in chlorobenzene (50% v/v) at 303 K in the absence or presence of compound **4d** or **11** (either 2.5×10^{-6} M), or of a mixture of **4d** (2.5×10^{-6} M) and one or two equivalents of **11**.

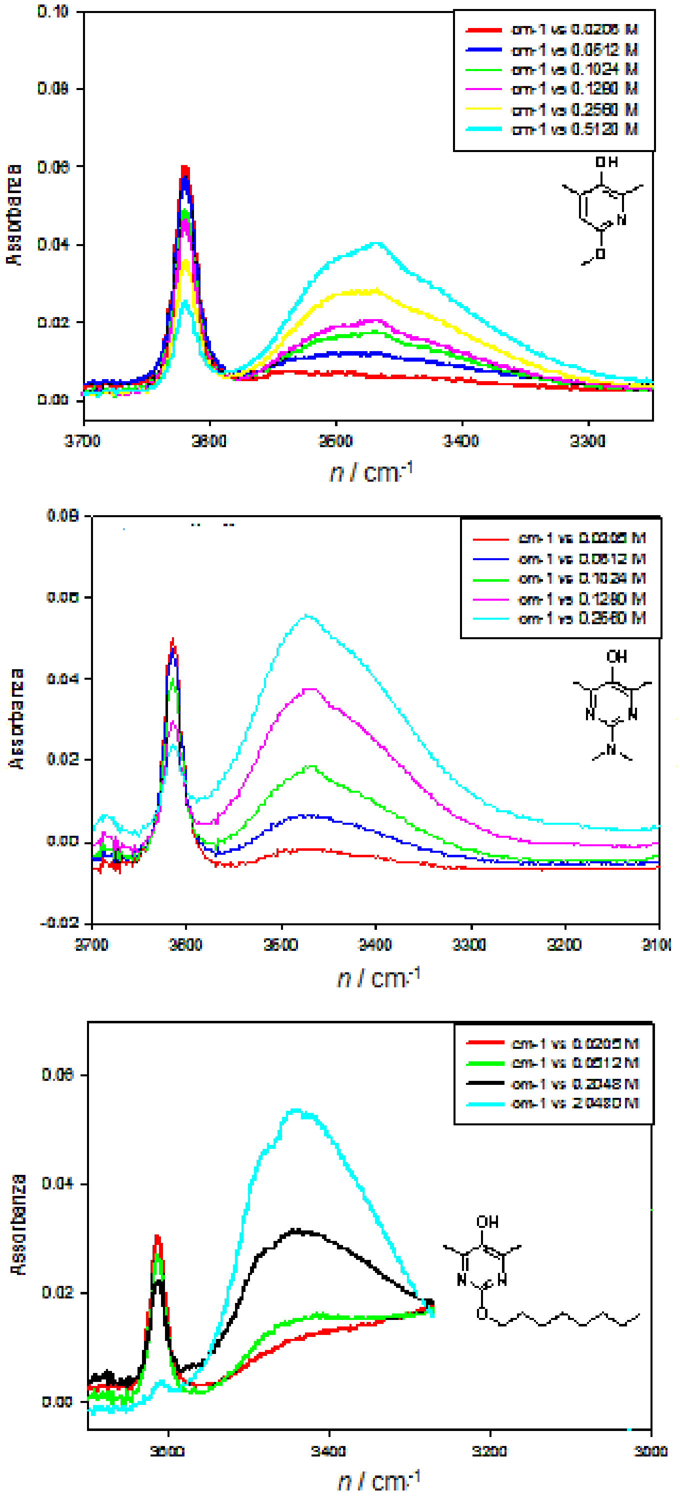

Figure S8: FTIR spectra recorded for compounds **5b**, **6b** and **7b** (10 mM) in CCl_4/EtOAc solvent mixtures.

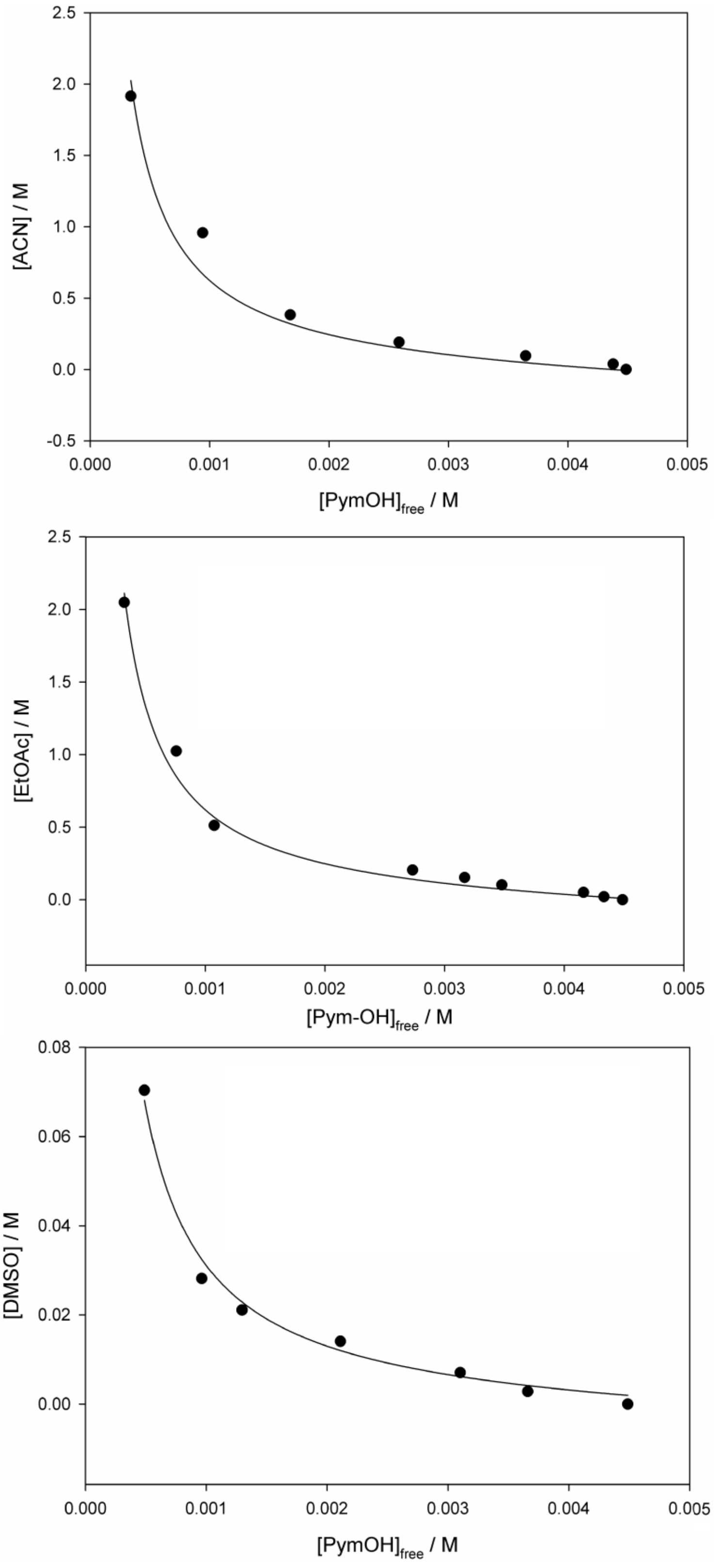

Figure S9: Plot of the concentration of HBA co-solvent (from left to right: acetonitrile, ethyl acetate, and dimethylsulfoxide) versus the concentration of "free" compound **7b** as determined by FTIR, fitted by equation 8 (see manuscript).

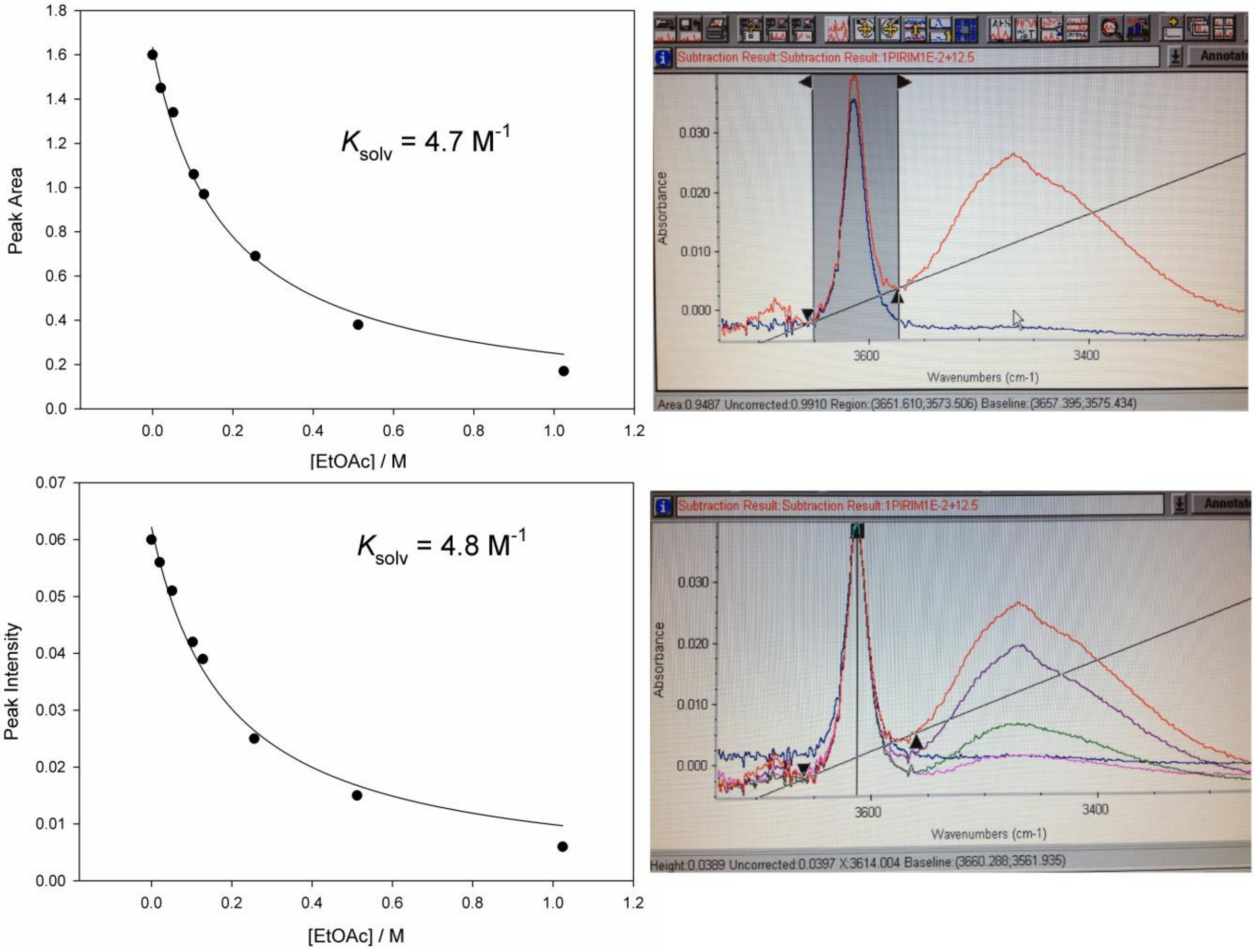

Figure S10: Treatment of IR data: the figure refers to IR spectra recorded for compound **6b** in solution (0.01M) using CCl_4/EtOAc mixtures with increasing concentration of the HBA co-solvent at 298 K. The top portion shows a plot of integrated peak area at 3610 cm^{-1} versus the concentration of EtOAc (left); adjacent is a sreenshot illustrating the procedure for manual baseline correction (right). The lower portion shows the corresponding plot obtained using peak height (left); adjacent is a corresponding screenshot illustrating the procedure (right). Data have been fit to equation 2 (see manuscript) and afford superimposable results.

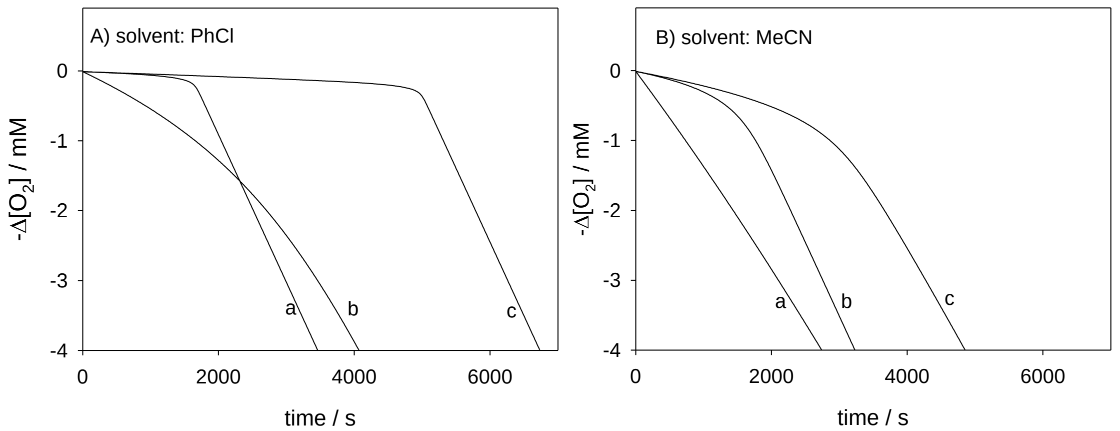

Figure S11: Simulated (Gepasi 3.0) oxygen consumption during the autoxidation of styrene (4.3 M) initiated by AIBN (0.05 M, $R_i = 6 \times 10^{-9}$ Ms^{-1}) at 30 °C in PhCl (A) or MeCN (B) and inhibited by: a) **4a** (5 μM); b) **11** (10 μM); c) **4a** + **11** (5 + 10 μM).

Table S1: Rate constants and corresponding α values calculated with Gepasi 3.0 software for the mixture **4a**/**11** protecting 4.3 M styrene in different solvents at 298 K. Data correspond to the plots shown in Figure S11 and have been obtained assuming $k_7 = 1 \times 10^8$ $M^{-1}s^{-1}$ (see manuscript).

	Kinetic rate constants/$M^{-1}s^{-1}$	
	PhCl	MeCN
k_{inh} (**4a**)	3.6×10^6	5.4×10^5
k_{inh} (**11**)	1.1×10^5	2.5×10^4
k_r	5.0×10^4	1.5×10^4
k_{-r}	9.3×10^2	1.4×10^2
α	1.0	0.46

Table S2: CBS-QB3-Calculated enthalpies at 298 K and minimum energy geometries of compounds **4**–**12**.

4° *H* = -456.68707 a.u.

C	0.59188800	0.01649000	0.07058900
C	-1.38563900	-1.17640700	0.03167100
C	-2.17086900	-0.02613200	-0.00903000
C	-1.50858800	1.19913200	-0.02833000
C	-0.12169700	1.23261200	0.00482300
H	-1.86888200	-2.14957600	0.03094000
H	-2.06939300	2.12872600	-0.07735700
H	0.38597800	2.18583100	-0.02488500
N	-0.05647300	-1.15951700	0.06649100
N	1.97637200	-0.02361900	0.16480600
C	2.67197700	-1.27243200	-0.11566900
H	2.86371900	-1.41214100	-1.19075500
H	3.62962800	-1.27441200	0.41027400
H	2.06749700	-2.10549200	0.23145200
C	2.73943400	1.19429200	-0.02314300
H	3.78918400	0.98483800	0.18461400
H	2.66807700	1.59834700	-1.04585500
H	2.41713500	1.97199000	0.67450300
O	-3.53478700	-0.16914200	-0.04103200
H	-3.94297200	0.70164900	-0.06923300

4a• *H* = -456.06546 a.u.

C	0.52843200	0.00982800	-0.00014200
C	-1.42988500	-1.19407700	-0.00014900
C	-2.27995100	-0.01464300	0.00001000
C	-1.55145900	1.23533700	0.00021100
C	-0.18559600	1.24801500	0.00014400
H	-1.92518700	-2.16241300	-0.00021500
H	-2.12658400	2.15427700	0.00046800
H	0.34786000	2.18925200	0.00040400
N	-0.11750400	-1.18188500	-0.00021700
N	1.89334600	-0.02309000	-0.00039200
C	2.63163100	-1.28200900	0.00043900
H	3.26805400	-1.34700900	-0.88830700
H	3.26648600	-1.34687300	0.89033900
H	1.92349700	-2.10423200	-0.00009600
C	2.67628100	1.20457100	-0.00018000
H	3.73290000	0.94252800	-0.00174700
H	2.47496000	1.81218600	-0.88850700
H	2.47715300	1.81064300	0.88973500
O	-3.52584500	-0.09445900	0.00002400

4b *H* = -535.15109 a.u.

C	0.99352900	0.04235300	-0.07676200
C	-0.95598000	-1.21192300	-0.02453100
C	-1.75007100	-0.06058200	-0.00230300
C	-1.13447700	1.19377000	0.00248500
C	0.25684100	1.24115000	-0.02901600
H	0.74719700	2.20441500	-0.01131600
N	0.37601500	-1.14886000	-0.05758400
N	2.38096400	0.03211500	-0.16891900
C	3.10369700	-1.19270900	0.14464800
H	4.06923700	-1.17893300	-0.36669700
H	3.28277500	-1.30951400	1.22493700
H	2.52693400	-2.04738000	-0.19684400
C	3.11438600	1.26997500	0.00343000
H	4.16971900	1.08259400	-0.19781500
H	2.77615300	2.02920100	-0.70683800
H	3.02999600	1.68839600	1.01965500
O	-3.11695500	-0.23374200	0.03056500
H	-3.54902700	0.62514900	0.03403100
C	-1.96137800	2.45432100	0.05018200
H	-2.62193200	2.53724600	-0.82131200
H	-2.59075600	2.49106900	0.94740400
H	-1.32626700	3.34061300	0.06132700
C	-1.59686900	-2.57369900	-0.00729700
H	-2.22603100	-2.70163700	0.87866100
H	-2.24824000	-2.71195200	-0.87564700
H	-0.82103700	-3.33805700	-0.01357600

4b● *H* = -534.53437 a.u.

C	0.94464000	0.03652100	-0.00823000
C	-0.98022500	-1.23814200	-0.00410000
C	-1.84512500	-0.05853800	-0.00109300
C	-1.16515600	1.22684100	0.00216200
C	0.20503400	1.25484500	-0.00079500
H	0.71918700	2.20748500	0.00672600
N	0.33561800	-1.17228100	-0.00716000
N	2.31225100	0.04002500	-0.01867500
C	3.08452500	-1.19657100	0.02645100
H	3.76075600	-1.24976400	-0.83297300
H	3.68248200	-1.23991000	0.94373400
H	2.39992300	-2.03806800	0.00254200
C	3.06258500	1.28702000	-0.01134800
H	4.12403900	1.05418100	-0.08024900
H	2.79868600	1.91984400	-0.86418600
H	2.89736500	1.85757500	0.90964100
O	-3.09089500	-0.16072300	0.00052000
C	-2.00849200	2.46471000	0.01112800
H	-2.66996100	2.48067400	-0.85979700
H	-2.66509300	2.47183200	0.88583500
H	-1.39861400	3.37010000	0.01394300
C	-1.63335200	-2.58650100	-0.00378300

H	-2.28038400	-2.69778500	0.87130100
H	-2.28601200	-2.69538100	-0.87494000
H	-0.87689700	-3.37030600	-0.00724200

4c H = -574.376546 a.u.

C	0.90568600	-0.10906800	-0.09555300
C	-1.03205800	-1.36840200	0.02911400
C	-1.81068300	-0.20882100	0.05788100
C	-1.20168200	1.04930900	-0.02424800
C	0.19489000	1.10978000	-0.14364300
N	0.29852600	-1.29499900	-0.02655400
N	2.32612600	-0.10579700	-0.16412200
C	2.93066200	-1.34519900	-0.64494000
H	3.98652800	-1.15268600	-0.85480900
H	2.85653200	-2.17028500	0.07729000
H	2.44159300	-1.66274000	-1.56531100
C	2.99438300	0.38326100	1.04323500
H	4.05317100	0.55285700	0.82910400
H	2.56160600	1.32812500	1.37089100
H	2.92187800	-0.33425000	1.87715600
O	-3.17109000	-0.37531300	0.15212200
H	-3.60612200	0.48134500	0.11299300
C	-2.07235100	2.28467400	-0.01434600
H	-2.61893400	2.40618700	-0.95769000
H	-2.81250100	2.23940400	0.79342100
H	-1.49389100	3.19224500	0.14571200
C	-1.67280900	-2.72882700	0.07429400
H	-2.27773800	-2.85173200	0.97776400
H	-2.34588100	-2.87769500	-0.77571600
H	-0.89480100	-3.49105000	0.05580900
C	0.91437900	2.41504700	-0.38386000
H	1.06597600	2.99139700	0.53665400
H	1.89461400	2.21331400	-0.81577400
H	0.36162300	3.05312700	-1.07733800

4c● H = -573.754761 a.u.

C	0.85403300	-0.17663300	-0.07718600
C	-1.12315500	-1.37067700	-0.00892800
C	-1.91796800	-0.15303400	0.12624400
C	-1.18733400	1.10300200	0.01225200
C	0.18480000	1.09376100	-0.13231800
N	0.19003800	-1.35241600	-0.09195900
N	2.23226700	-0.28011600	-0.06813100
C	2.84589000	-1.56988800	-0.36978200
H	3.87187900	-1.38909800	-0.70132200
H	2.86950800	-2.22929900	0.50688400
H	2.29093000	-2.07287400	-1.15655000
C	3.06095700	0.56704700	0.78544100
H	3.91696700	0.96595500	0.23238100
H	2.48904700	1.39312300	1.19502100
H	3.44436900	-0.02389100	1.62774000

O	-3.15664200	-0.20635100	0.28027000
C	-2.00148700	2.36605900	-0.00613300
H	-2.04801100	2.79386800	-1.01474900
H	-3.01990700	2.13877100	0.30569000
H	-1.58211300	3.13176100	0.65191100
C	-1.83418700	-2.68858000	-0.03435100
H	-2.45824200	-2.80421200	0.85611500
H	-2.51642000	-2.74071400	-0.88868900
H	-1.11113000	-3.50136600	-0.09142800
C	0.92286900	2.37413000	-0.45883500
H	1.13728300	2.98344700	0.42638300
H	1.86740300	2.16981500	-0.96328500
H	0.31893400	2.99213400	-1.12605400

4d *H* = -535.162191 a.u.

C	1.16671300	0.05283300	0.02088400
C	-0.62520900	-1.40160300	0.02236200
C	-1.52193800	-0.33623400	-0.00284700
C	-1.04887800	0.98644900	-0.02062700
C	0.33308700	1.19851900	0.02088900
N	0.69624300	-1.19385500	0.01861700
C	3.41023300	-0.96256200	-0.08600800
H	3.27552300	-1.47042700	-1.04983100
H	4.44843900	-0.63799300	0.00814600
H	3.19690800	-1.68984100	0.69711100
O	-2.86505600	-0.64865600	-0.02786700
H	-3.38471500	0.14855300	0.10627900
C	-2.06073400	2.10809100	-0.07731000
H	-2.81988200	1.90861600	-0.84190100
H	-2.58040900	2.23671000	0.88043300
H	-1.60948700	3.06478700	-0.33092300
C	-1.11827000	-2.82299300	0.03808900
H	-1.76440400	-3.00704400	0.90162500
H	-1.71533000	-3.04470600	-0.85196300
H	-0.26435700	-3.49839800	0.07433500
C	0.97238700	2.56369900	0.08705500
H	1.75859400	2.57368100	0.84850600
H	1.44113300	2.84750000	-0.86488100
H	0.26353400	3.34937800	0.34355000
N	2.55093300	0.20133500	0.07445300
H	2.90032500	1.03888000	-0.36395900

4d• *H* = -534.54606 a.u.

C	1.11761800	0.06223500	-0.00228100
C	-0.61346100	-1.44812300	-0.00150200
C	-1.61146700	-0.38385900	0.00122600
C	-1.10588800	0.98713400	0.00091100
C	0.25185400	1.20722400	-0.00147400
N	0.68560400	-1.21301000	-0.00254200
C	3.41178800	-0.87858600	0.00676600
H	3.25816600	-1.53219700	-0.85429300

H	4.42164700	-0.46887300	-0.02758900
H	3.29943800	-1.48520000	0.90885900
O	-2.83333000	-0.65225800	0.00332100
C	-2.12994000	2.08972000	0.00293200
H	-3.12464500	1.64683700	0.00610800
H	-2.03163100	2.73084000	0.88457500
H	-2.03684800	2.72909300	-0.88061200
C	-1.08799900	-2.86831900	-0.00280400
H	-1.71976800	-3.06281200	0.86886500
H	-1.71567500	-3.06246800	-0.87758900
H	-0.23494100	-3.54577300	-0.00123400
C	0.86109000	2.58832300	-0.00368200
H	1.48499200	2.75403700	0.88312300
H	1.49342500	2.74666300	-0.88563300
H	0.09818800	3.36388000	-0.01012500
N	2.46855600	0.22811100	-0.00554900
H	2.83360800	1.16383500	0.01505500

5a H = -437.340388 a.u.

C	0.89494700	0.30641600	0.00002800
C	-0.85155900	-1.17193500	-0.00010800
C	-1.79542300	-0.15135300	-0.00003500
C	-1.33266600	1.16965200	-0.00004700
C	0.02959200	1.41021600	-0.00005100
H	-1.18028100	-2.20641100	-0.00017200
H	-2.03423600	1.99911800	-0.00009400
H	0.43524500	2.41334300	-0.00018900
N	0.46795600	-0.94621200	-0.00001300
C	3.10667900	-0.55917500	-0.00000400
H	4.11037500	-0.13711600	0.00015400
H	2.95404500	-1.17865000	0.88679600
H	2.95412300	-1.17844800	-0.88691200
O	-3.12074300	-0.49383300	0.00009500
H	-3.65666600	0.30532100	0.00031300
O	2.22477900	0.56675800	0.00009300

5a● H = -436.711583 a.u.

C	0.82815300	0.29826000	-0.00000900
C	-0.88153500	-1.20219000	0.00023300
C	-1.90054000	-0.16833100	-0.00000900
C	-1.39247100	1.19187700	0.00011500
C	-0.04913700	1.41876900	0.00011600
H	-1.21544700	-2.23616500	0.00043700
H	-2.11410400	2.00028600	0.00031600
H	0.37955200	2.41335300	0.00025700
N	0.42144300	-0.97232000	0.00017500
C	3.05815100	-0.53437800	-0.00009100
H	4.04470500	-0.07653000	-0.00012500
H	2.91743300	-1.15111500	0.88829300
H	2.91750000	-1.15136200	-0.88827100
O	-3.11782200	-0.44755000	-0.00033400

O	2.13589000	0.57051700	-0.00019800

5b *H* = -515.804474 a.u.

C	-1.21345300	-0.48918000	0.00002800
C	0.33584900	1.20640400	0.00004300
C	1.38434400	0.28569800	0.00000500
C	1.10703500	-1.09107400	-0.00000800
C	-0.22558800	-1.47947900	0.00000400
H	-0.51234800	-2.52361800	-0.00003600
N	-0.94612100	0.80626200	0.00005400
C	-3.51746400	0.09732900	-0.00007900
H	-4.46150500	-0.44570700	-0.00007800
H	-3.44444700	0.73142700	0.88671400
H	-3.44433900	0.73130500	-0.88695200
O	2.66405900	0.79023900	-0.00003900
H	3.29730400	0.06665900	0.00025800
O	-2.50393400	-0.90996300	0.00007200
C	2.22705600	-2.10020200	-0.00003500
H	2.86564100	-1.99457200	-0.88509000
H	2.86563800	-1.99460200	0.88502500
H	1.83633400	-3.11788000	-0.00004500
C	0.61391500	2.68419500	-0.00000800
H	1.20010400	2.97507800	0.87692400
H	1.19881200	2.97525600	-0.87775600
H	-0.32951500	3.22846100	0.00069800

5b• *H* = -515.18128 a.u.

C	-1.15496300	-0.49660500	0.00020300
C	0.32358900	1.24505300	-0.00135400
C	1.46908000	0.33573400	-0.00099800
C	1.17127700	-1.09505500	0.00044500
C	-0.13850800	-1.48595800	0.00108400
H	-0.43265500	-2.52911900	0.00198600
N	-0.93400700	0.81696600	-0.00098400
C	-3.48130400	0.01546300	-0.00061400
H	-4.39489400	-0.57525200	0.00065100
H	-3.42961200	0.64806100	0.88647600
H	-3.43046200	0.64541400	-0.88964300
O	2.63943700	0.77096700	-0.00173800
O	-2.41548300	-0.94894400	0.00030100
C	2.32135100	-2.05285100	0.00057800
H	2.95620400	-1.88468300	-0.87385600
H	2.95846400	-1.88171600	0.87276700
H	1.98100800	-3.08937500	0.00266500
C	0.58123700	2.71801400	0.00115200
H	1.13006700	3.00918300	0.90226200
H	1.21892700	2.99801200	-0.84199500
H	-0.36118600	3.26175800	-0.04590700

6a *H* = -472.738328 a.u.

C	0.55436500	0.00166600	0.00352700
C	-1.39544900	-1.19084400	0.00032600
C	-2.14377100	-0.01374000	-0.00023500
C	-1.40867500	1.16661000	0.00010000
H	-1.90085500	-2.15326700	-0.00106600
H	-1.91087900	2.13471100	-0.00175800
N	-0.06994000	-1.19604200	0.00187800
N	1.92606100	0.00546400	0.01009100
C	2.69804900	-1.22350600	-0.00415900
H	3.31923200	-1.27777300	-0.90731200
H	3.36356800	-1.26595500	0.86680500
H	2.02536800	-2.07528000	0.01746300
C	2.68919200	1.23960000	-0.00345600
H	3.30617600	1.30172400	-0.90903100
H	2.01080600	2.08678500	0.02399600
H	3.35818900	1.28377600	0.86474500
O	-3.51173200	-0.08898300	-0.00259100
H	-3.87783600	0.80151700	-0.00340900
N	-0.07928800	1.19013700	0.00141300

6a• *H* = -472.116107 a.u.

C	0.49353400	0.00000200	0.00009700
C	-1.44093500	-1.20554600	0.00010500
C	-2.24407100	-0.00001600	-0.00003000
C	-1.44093000	1.20553600	0.00014600
H	-1.95365200	-2.16506000	0.00016400
H	-1.95369300	2.16502600	0.00023200
N	-0.12987500	-1.20774500	0.00016200
N	1.85286200	0.00000500	-0.00003300
C	2.62651300	-1.23414700	-0.00008300
H	3.26712800	-1.26948200	-0.88763000
H	3.26719100	-1.26953500	0.88742000
H	1.95310800	-2.08453300	-0.00006400
C	2.62650500	1.23414500	-0.00013800
H	3.26711600	1.26945400	-0.88769300
H	1.95310400	2.08453500	-0.00015900
H	3.26719900	1.26956700	0.88735600
O	-3.49286800	0.00000800	-0.00030700
N	-0.12988000	1.20775700	0.00019400

6b *H* = -551.207061 a.u.

C	0.94444000	0.00178200	0.05195100
C	-1.00434300	-1.20582700	0.00882300
C	-1.73353800	-0.01122300	0.00258900
C	-1.01631500	1.18438700	0.00611700
N	0.32676800	-1.19508700	0.02964500
N	2.31949200	0.00546000	0.12651400
C	3.07214800	-1.22342100	-0.05871800
H	3.35943000	-1.37375500	-1.11017200
H	3.98547000	-1.17904600	0.54114200

H	2.47148700	-2.07126200	0.25721100
C	3.06147300	1.24262600	-0.04257600
H	3.32279100	1.42529500	-1.09577600
H	2.46606600	2.07924800	0.31166800
H	3.98889900	1.18499100	0.53373400
O	-3.10775100	-0.08902200	-0.02262900
H	-3.47829000	0.79846100	-0.01540700
N	0.31915600	1.19083800	0.02670000
C	-1.72038100	2.51707700	-0.01953100
H	-2.35844000	2.61998500	-0.90523600
H	-2.35205000	2.65644800	0.86586000
H	-0.97945100	3.31431700	-0.03801200
C	-1.70929500	-2.53214100	-0.01350700
H	-2.35483200	-2.64222600	0.86291500
H	-2.35762700	-2.61274800	-0.89078700
H	-0.97448400	-3.33556700	-0.02701400

6b• *H* = -550.589069 a.u.

C	0.89458800	-0.00001300	0.00015400
C	-1.03923100	-1.22422100	0.00012200
C	-1.82741300	-0.00000700	-0.00021100
C	-1.03920100	1.22422800	0.00015300
N	0.27764100	-1.20744200	0.00031300
N	2.25676500	0.00000100	-0.00001900
C	3.03125200	-1.23254300	-0.00018900
H	3.67230600	-1.26894700	-0.88783000
H	3.67295500	-1.26873600	0.88699200
H	2.35815300	-2.08309700	0.00016600
C	3.03120500	1.23254900	-0.00022700
H	3.67233500	1.26888200	-0.88782100
H	2.35807500	2.08308100	-0.00002700
H	3.67284400	1.26887100	0.88700100
O	-3.07803700	0.00001700	-0.00077500
N	0.27765700	1.20743400	0.00025400
C	-1.75485500	2.53772600	0.00028900
H	-2.40919000	2.61292800	-0.87311800
H	-2.40972300	2.61242800	0.87333500
H	-1.03892800	3.35842100	0.00072500
C	-1.75486600	-2.53773400	0.00028300
H	-2.41070300	-2.61195400	0.87263000
H	-2.40822300	-2.61345800	-0.87382300
H	-1.03891900	-3.35841000	0.00188700

7a *H* = -531.85515 a.u.

C	-1.16865900	-0.46648000	0.00002600
C	0.36838500	1.21546400	-0.00000600
C	1.38296700	0.25689900	0.00000500
C	1.01111400	-1.09256100	-0.00000800
N	-0.91700800	0.83980700	0.00000300
C	-3.46963600	0.11403900	-0.00003900
H	-4.40800500	-0.43805700	0.00000500

H	-3.40201500	0.74752100	0.88726100
H	-3.40197700	0.74736800	-0.88743700
O	2.68436900	0.69214100	0.00002800
H	3.27795400	-0.06500800	0.00009200
N	-0.27338700	-1.45276400	0.00000200
C	2.04400700	-2.18880200	-0.00003700
H	2.68778000	-2.13697900	-0.88607400
H	2.68777400	-2.13706300	0.88601100
H	1.53877600	-3.15281500	-0.00007400
C	0.68724500	2.68185800	-0.00002600
H	1.28308000	2.94988800	0.87739100
H	1.28412000	2.94965100	-0.87679600
H	-0.23808600	3.25571800	-0.00060600
O	-2.44801500	-0.88614500	0.00005900

7a● *H* = -531.228775 a.u.

C	-1.11125400	-0.47725700	0.00001900
C	0.36383000	1.25374500	-0.00182100
C	1.46642900	0.30508800	-0.00060200
C	1.06956800	-1.10188100	0.00065000
N	-0.89687200	0.84553100	-0.00186100
C	-3.43172600	0.04400800	-0.00023200
H	-4.34368100	-0.54877900	0.00210700
H	-3.38063700	0.67653600	0.88676900
H	-3.38278000	0.67239400	-0.89031600
O	2.66315000	0.66550300	-0.00097900
N	-0.19565100	-1.46535000	0.00114900
C	2.13169500	-2.15235000	0.00028900
H	2.77665500	-2.03924400	-0.87604200
H	2.78280200	-2.03391800	0.87126400
H	1.67712000	-3.14142900	0.00448300
C	0.65891200	2.71749600	0.00069100
H	1.20505800	2.99214000	0.90857200
H	1.31553200	2.97508600	-0.83483300
H	-0.26683300	3.28763500	-0.05811400
O	-2.36568700	-0.91985200	0.00062000

7b *H* = -571.084655 a.u.

C	-0.75536800	-0.34314700	-0.00002700
C	0.91098800	1.21149600	-0.00001400
C	1.84665200	0.17556100	0.00000600
C	1.36870200	-1.13992000	-0.00000100
N	-0.40019900	0.93937700	-0.00003000
C	-3.01938000	0.41463600	-0.00000400
H	-2.86004000	1.04233600	0.88131300
H	-2.86008200	1.04231300	-0.88134600
O	3.17866300	0.50599100	0.00002700
H	3.70996000	-0.29610000	0.00003400
N	0.05971000	-1.39710200	-0.00001300
C	2.31166500	-2.31454800	0.00000400
H	2.95743700	-2.31370900	-0.88605000

H	2.95738800	-2.31374100	0.88609300
H	1.73177400	-3.23559200	-0.00002800
C	1.34607400	2.64773800	-0.00000400
H	1.96172700	2.86681300	0.87732700
H	1.96265900	2.86656300	-0.87673300
H	0.46998000	3.29423400	-0.00052700
O	-2.06337300	-0.66041500	-0.00001400
C	-4.39750200	-0.21719500	0.00003600
H	-5.16501500	0.56142100	0.00004200
H	-4.53785000	-0.84141000	-0.88511600
H	-4.53780900	-0.84138500	0.8852130

7b● H = -570.458542 a.u.

C	-0.70628500	-0.33290000	-0.00008000
C	0.93266900	1.24479900	-0.00156400
C	1.93725000	0.19277300	-0.00130200
C	1.40402900	-1.16819000	0.00066100
N	-0.36199700	0.96300500	-0.00148300
C	-2.98210800	0.41110600	-0.00141100
H	-2.82604700	1.03747900	0.87940500
H	-2.82724000	1.03386900	-0.88500700
O	3.16361800	0.43414300	-0.00204900
N	0.10939800	-1.40573900	0.00131400
C	2.35808800	-2.31786800	0.00099500
H	3.01137500	-2.26891800	-0.87510500
H	3.01750600	-2.26351300	0.87210000
H	1.80867100	-3.25759600	0.00547700
C	1.37089900	2.67238600	0.00160100
H	1.94534500	2.88987400	0.90748000
H	2.04647500	2.86519800	-0.83631600
H	0.50605100	3.33159300	-0.05281700
O	-1.99684000	-0.65080900	0.00003200
C	-4.34409000	-0.25137700	0.00078400
H	-5.12494700	0.51337100	-0.00030500
H	-4.47351800	-0.87845100	-0.88354400
H	-4.47241500	-0.87480700	0.88784500

8 H = -704.558006

C	-0.13197000	0.00072100	0.00049400
C	1.80600500	1.14725200	-0.38137900
C	2.53067000	0.00950400	-0.00389200
C	1.81446100	-1.12725300	0.37597300
N	-1.54258700	-0.00216900	0.00008300
C	-2.35449100	-1.08831500	-0.36482500
C	-2.35869700	1.08058100	0.36583000
C	-3.65413200	-0.68059000	-0.23159300
C	-3.65671300	0.66709800	0.23413000
H	-4.51664600	-1.28853600	-0.45985600
H	-4.52152100	1.27164400	0.46265300
N	0.47757300	-1.11766400	0.38978800
N	0.47250400	1.12523300	-0.38965600

C	2.50995500	2.40514000	-0.79920500
H	3.10581700	2.80710600	0.02589400
H	3.20508900	2.20723600	-1.61977200
H	1.77700400	3.14692600	-1.11150800
C	2.51563100	-2.39099900	0.79960200
H	3.10749500	-2.81725200	-0.01897800
H	3.19049100	-2.21411700	1.64526700
H	1.77530800	-3.12821400	1.10450700
O	3.89593900	0.08074700	-0.02776100
H	4.27215600	-0.76532800	0.23514000
C	-1.84383200	-2.39859100	-0.87144300
H	-1.13892200	-2.27630500	-1.70021300
H	-1.33216300	-2.97402800	-0.09763300
H	-2.69181800	-2.98029400	-1.23867300
C	-1.85319300	2.39375200	0.87015300
H	-1.14872800	2.27589800	1.69995000
H	-1.34199700	2.96839400	0.09570300
H	-2.70366500	2.97329200	1.23503700

8• *H* = -703.930117 a.u.

C	-0.08870300	0.00001700	-0.00004600
C	1.83736400	-1.20061200	0.25588300
C	2.62500700	0.00001500	0.00005200
C	1.83741100	1.20059600	-0.25594100
N	-1.47928900	0.00001100	-0.00002800
C	-2.30712900	1.13241400	0.22418900
C	-2.30712000	-1.13239800	-0.22420100
C	-3.59638300	0.70080300	0.14587700
C	-3.59638000	-0.70084000	-0.14567000
H	-4.46238200	1.32653600	0.29931100
H	-4.46237800	-1.32660900	-0.29896300
N	0.51950500	1.17505700	-0.25470500
N	0.51948100	-1.17509600	0.25458200
C	2.54852600	-2.48612500	0.52934100
H	3.13730800	-2.78541600	-0.34360500
H	3.26450900	-2.35848600	1.34570000
H	1.83316900	-3.26902200	0.77536700
C	2.54854600	2.48612500	-0.52935800
H	3.13569400	2.78643200	0.34436100
H	3.26598100	2.35806700	-1.34435900
H	1.83330500	3.26857000	-0.77716000
O	3.87213400	-0.00003100	0.00010700
C	-1.83879100	2.50878400	0.57282200
H	-1.14483500	2.51148700	1.41735400
H	-1.33087400	3.00122800	-0.25694300
H	-2.71507100	3.09787300	0.85213500
C	-1.83878500	-2.50874200	-0.57294600
H	-1.14488100	-2.51137600	-1.41751900
H	-1.33080400	-3.00123100	0.25675700
H	-2.71507000	-3.09783000	-0.85224100

9a *H* = -651.65762 a.u.

C	1.44232900	1.17303900	0.05174800
C	0.07444300	0.88847600	0.05089800
C	-0.33550300	-0.46562200	-0.01026200
C	1.86300700	-1.19802600	-0.07444900
C	2.34811500	0.10702200	-0.01945200
C	-2.31128600	1.51971300	-0.37470200
H	-3.07845500	2.26693600	-0.15330600
H	-2.28901100	1.38730200	-1.46215200
C	-0.94919900	1.99677700	0.11862800
H	-0.62045100	2.85448100	-0.47530800
H	-1.03643300	2.36219600	1.15108900
C	1.95360600	2.59024400	0.13149500
H	1.71897200	3.14654100	-0.78259800
H	1.49564200	3.13129500	0.96422000
H	3.03351800	2.60437500	0.26263500
C	2.80572500	-2.37554900	-0.14293900
H	2.22480000	-3.29599900	-0.17037900
H	3.43458900	-2.34548900	-1.04149500
H	3.47071000	-2.42109000	0.72879500
C	-4.09863100	-0.23297600	-0.22258000
H	-4.48326600	-1.10534700	0.30720800
H	-4.80218000	0.58695900	-0.05797300
H	-4.07752000	-0.45917000	-1.29263700
C	-2.03249000	-2.20178500	0.31228400
H	-1.17622300	-2.83589400	0.10796200
H	-2.28790200	-2.29226900	1.38042300
H	-2.88008800	-2.55428300	-0.27661200
N	-1.69223000	-0.82864700	-0.04690600
O	3.69657800	0.39593000	-0.02683100
H	4.19326400	-0.42663400	-0.05966500
C	-2.70738400	0.18700000	0.26371800
H	-2.76094900	0.32957800	1.35963200
N	0.55393900	-1.46147000	-0.06474000

9a● *H* = -651.040834 a.u.

C	1.46655700	1.19935100	0.05091700
C	0.12174700	0.91700600	0.12143200
C	-0.29856200	-0.45256200	0.08416800
C	1.86781900	-1.23663900	-0.08579200
C	2.42752600	0.11131900	-0.06963400
C	-2.23998500	1.54185700	-0.36085400
H	-3.02443800	2.28529200	-0.19664500
H	-2.11551500	1.43609800	-1.44456600
C	-0.92453400	1.99474600	0.26324700
H	-0.58306600	2.91958400	-0.20592100
H	-1.07860300	2.22785400	1.32583200
C	2.01391100	2.59876900	0.10802100
H	1.67436700	3.19910600	-0.74312900
H	1.69972500	3.11923200	1.01812800
H	3.10176600	2.55478700	0.08474300

C	2.81329700	-2.39399500	-0.19272900
H	2.25800100	-3.33104400	-0.21410000
H	3.42741200	-2.30436900	-1.09342300
H	3.51323300	-2.39638900	0.64831000
C	-3.96812100	-0.27126100	-0.51798000
H	-4.41247500	-1.15665100	-0.06215400
H	-4.71819300	0.52277800	-0.49274000
H	-3.74306500	-0.49559200	-1.56429000
C	-2.01012400	-2.18041900	0.43416900
H	-1.10937000	-2.76508800	0.58583500
H	-2.63237600	-2.21643200	1.33588100
H	-2.57407900	-2.61437600	-0.39528200
N	-1.62935200	-0.79756900	0.15373600
O	3.66154800	0.29749700	-0.14705500
C	-2.70846300	0.20711700	0.21550700
H	-2.96738800	0.35567100	1.27678900
N	0.57582100	-1.47845600	-0.00940200

9b *H* = -690.887874 a.u.

C	1.60935200	1.18515900	-0.02288400
C	0.24969100	0.87722800	-0.10991300
C	-0.13509200	-0.48562700	-0.14592900
C	2.07301600	-1.18128100	-0.02337600
C	2.53366200	0.13301500	0.01165600
C	-2.53671500	0.11024700	0.04928800
C	-2.14179400	1.44108800	-0.62169300
H	-2.92245700	2.18359800	-0.43275400
H	-2.11160400	1.27002100	-1.70250300
C	-0.78958400	1.97012700	-0.15261900
H	-0.46556900	2.77586700	-0.81874200
H	-0.88668600	2.43150100	0.83924100
C	2.09023800	2.61381100	0.03128800
H	1.89426800	3.13197100	-0.91378000
H	1.57708700	3.17538200	0.81729000
H	3.16085800	2.65524200	0.21992100
C	3.03496200	-2.34424600	0.02362600
H	2.46842200	-3.27401400	0.01610000
H	3.71171900	-2.35197100	-0.83986400
H	3.65193200	-2.33218600	0.93121100
C	-3.88063200	-0.32742600	-0.55880700
H	-4.31411400	-1.18764800	-0.04552600
H	-4.59719500	0.49354200	-0.47610500
H	-3.76441200	-0.57453300	-1.61705700
C	-2.69790300	0.27926000	1.57622400
H	-3.45558700	1.03466800	1.80622600
H	-3.01365800	-0.66067100	2.03603100
H	-1.76006800	0.58094200	2.04713300
C	-1.76355400	-2.29293900	-0.07244500
H	-1.01022700	-2.88069600	-0.58936900
H	-1.74245200	-2.59285100	0.98581700
H	-2.74050600	-2.53304700	-0.48661800

N	-1.47542800	-0.87542300	-0.26848000
O	3.87506700	0.44549300	0.08656100
H	4.38394100	-0.36887800	0.13370900
N	0.76998500	-1.46895900	-0.08999800

9b• H = -690.270891 a.u.

C	1.64357400	1.21189900	0.00084600
C	0.30383900	0.89655400	-0.04107200
C	-0.08027100	-0.48486900	-0.05207800
C	2.11712200	-1.21079800	-0.02341800
C	2.63941100	0.15109900	0.01178300
C	-2.51464300	0.11762100	0.00300500
C	-2.05532300	1.42580100	-0.66369000
H	-2.85712900	2.16363800	-0.57388500
H	-1.91290800	1.23295300	-1.73228700
C	-0.75866100	1.96695600	-0.07414900
H	-0.41320400	2.81703200	-0.66763100
H	-0.93094400	2.35949800	0.93591700
C	2.14471000	2.62871800	0.03382100
H	1.90184000	3.16169100	-0.89245400
H	1.70288500	3.19653000	0.85855100
H	3.22766300	2.61990600	0.14806000
C	3.09729600	-2.34419400	-0.02406200
H	2.56975900	-3.29690400	-0.05443600
H	3.77244800	-2.26589400	-0.88129900
H	3.73458000	-2.30005700	0.86409800
C	-3.73551800	-0.40366700	-0.77586300
H	-4.18085400	-1.28744000	-0.31647600
H	-4.50094400	0.37547800	-0.79826500
H	-3.46810300	-0.64540100	-1.80741000
C	-2.90313300	0.35012400	1.47893000
H	-3.70952800	1.08554800	1.54835600
H	-3.25642400	-0.57428600	1.94172500
H	-2.05529500	0.71180300	2.06300700
C	-1.74715800	-2.28630800	0.05294700
H	-0.83433200	-2.84960700	0.21125400
H	-2.42334500	-2.44438900	0.89791900
H	-2.23414400	-2.65089400	-0.85533200
N	-1.40231400	-0.86968400	-0.06743900
O	3.87034100	0.37019300	0.04670400
N	0.83056700	-1.48522600	-0.05042400

9c H = -726.797496 a.u.

C	1.56256700	1.18940300	-0.03754700
C	0.21682800	0.84063700	-0.13441700
C	-0.13737700	-0.52576700	-0.15509400
C	2.07811900	-1.17004100	-0.01431200
C	2.50799700	0.15645600	0.02019300
C	-2.48351800	0.12502400	0.05912500
C	-0.86255800	1.88635900	-0.22671900
H	-0.63056800	2.61557600	-1.00734100

H	-0.94803100	2.44759700	0.71771100
C	1.99896800	2.63262200	-0.01156200
H	1.75347700	3.13324800	-0.95436800
H	1.49413600	3.18534300	0.78679500
H	3.07285200	2.71191400	0.14240500
C	3.06858700	-2.30783000	0.04389500
H	2.52668800	-3.25193900	0.02444000
H	3.75835900	-2.29508800	-0.80913600
H	3.67062100	-2.28264500	0.96100500
C	-3.84219800	-0.21742500	-0.55045200
H	-4.31188500	-1.07125500	-0.06119000
H	-4.49412000	0.64922400	-0.43459400
H	-3.72621300	-0.42498900	-1.61529000
C	-2.60006700	0.33363100	1.58059000
H	-3.30386100	1.14134300	1.79537700
H	-2.96332500	-0.57773100	2.06120100
H	-1.63491300	0.58155200	2.02628700
C	-1.81760000	-2.30680000	-0.10037700
H	-1.00664000	-2.90759300	-0.50228300
H	-1.95097400	-2.59191900	0.95406800
H	-2.73239200	-2.53784300	-0.64490300
N	-1.47560300	-0.90097100	-0.27027400
O	3.83950800	0.50089400	0.10966100
H	4.36972100	-0.30018200	0.14945000
N	0.78212000	-1.49218300	-0.09007500
O	-2.11788800	1.33783700	-0.59805200

9c• *H* = -726.179839 a.u.

C	1.59242900	1.22121800	-0.01944400
C	0.26723600	0.86333000	-0.08235700
C	-0.08361900	-0.52145000	-0.07901000
C	2.12197000	-1.19501200	-0.01240700
C	2.61058400	0.18184900	0.02760600
C	-2.46521300	0.13092100	0.02692100
C	-0.83405300	1.88581900	-0.16999800
H	-0.58608300	2.65737900	-0.90090600
H	-0.98732500	2.39169500	0.79471500
C	2.04784800	2.65297000	-0.01309700
H	1.76976400	3.16452400	-0.94146900
H	1.60295800	3.21717100	0.81312800
H	3.13206400	2.68222600	0.08472500
C	3.13085800	-2.30198900	0.00686400
H	2.62823900	-3.26812200	-0.01795900
H	3.81033100	-2.21539300	-0.84624700
H	3.75967400	-2.23174400	0.89919700
C	-3.72386900	-0.29037000	-0.73031800
H	-4.20163600	-1.16370500	-0.28639000
H	-4.42742900	0.54224800	-0.70330800
H	-3.47666000	-0.49893000	-1.77216300
C	-2.76659300	0.39174700	1.51330600
H	-3.51253600	1.18434900	1.60357700

H	-3.16051300	-0.50861700	1.98944600
H	-1.86825900	0.68854600	2.05701300
C	-1.77545300	-2.30902800	-0.01677400
H	-0.86497500	-2.89574500	0.05071800
H	-2.39132800	-2.49734100	0.86844700
H	-2.33451300	-2.61545000	-0.90403600
N	-1.40118500	-0.89985600	-0.09744900
O	3.83394900	0.43130100	0.08798200
N	0.84238400	-1.50453600	-0.05971900
O	-2.04806400	1.31090100	-0.63749100

10 *H* = -659.968829 a.u.

C	0.00943600	2.27994300	-0.00862800
C	-1.18332300	1.56638200	-0.00785600
C	-1.22757400	0.16667900	-0.00044000
C	0.00837300	-0.51473800	0.00464700
C	1.24734200	0.16607900	0.00011500
C	1.20407900	1.56051500	-0.00654300
H	-2.10792900	2.12708100	-0.01441500
H	2.13112500	2.11811000	-0.01203800
C	-2.58773300	-0.57729100	-0.00083400
C	2.59594000	-0.59102400	-0.00087000
C	0.01733800	3.79097300	0.01010100
H	0.26347000	4.17763600	1.00536600
H	0.75739700	4.19620100	-0.68580800
H	-0.95819500	4.19728900	-0.26675200
C	2.72463800	-1.46437600	1.27091200
H	1.94058100	-2.21706600	1.32471300
H	3.69317100	-1.97466400	1.27418100
H	2.66983000	-0.84323300	2.16984700
C	3.79604300	0.37839500	-0.00709900
H	3.80715600	1.01439700	-0.89642700
H	3.81119700	1.02082200	0.87753300
H	4.72227800	-0.20218800	-0.00716800
C	2.71824500	-1.47291900	-1.26738800
H	2.65896000	-0.85776000	-2.17014600
H	3.68669700	-1.98335300	-1.27214200
H	1.93386200	-2.22585900	-1.31213500
C	-3.77941100	0.40401200	-0.00746400
H	-3.78046800	1.03872300	-0.89661200
H	-4.71272100	-0.16519600	-0.00694900
H	-3.78404800	1.04636500	0.87616000
C	-2.75055900	-1.43616000	1.28126200
H	-1.98980300	-2.20992000	1.40982800
H	-2.69980800	-0.79954700	2.16788500
H	-3.72332600	-1.93670600	1.27680900
C	-2.74478000	-1.44682700	-1.27643200
H	-3.71747600	-1.94752100	-1.27218100
H	-2.69015400	-0.81760100	-2.16808900
H	-1.98330200	-2.22149900	-1.39511400
O	0.07432400	-1.89177900	0.01041100

H	-0.81140700	-2.25813600	0.00946600

10• H = -659.345993 a.u.

C	0.00007300	2.25495700	-0.01478400
C	-1.21651700	1.54527200	-0.01267000
C	-1.27232000	0.16850300	-0.00194200
C	-0.00018000	-0.58066600	0.00742200
C	1.27228100	0.16826000	-0.00185400
C	1.21683800	1.54468300	-0.01248300
H	-2.13145400	2.12372900	-0.02110600
H	2.13194200	2.12297500	-0.02075200
C	-2.60461600	-0.59479000	-0.00167300
C	2.60427000	-0.59555800	-0.00167700
C	0.00152100	3.75840400	0.01522400
H	0.03278100	4.12317300	1.04954300
H	0.87419600	4.16921900	-0.49777300
H	-0.89845300	4.17057300	-0.44633500
C	2.71011900	-1.47125400	1.27229800
H	1.91433200	-2.21250300	1.30587600
H	3.67363200	-1.99006700	1.28139600
H	2.65355700	-0.85361000	2.17376300
C	3.81398700	0.35976100	-0.01547200
H	3.82989200	0.99154700	-0.90819500
H	3.83865600	1.00609300	0.86656400
H	4.73421400	-0.22964700	-0.01515100
C	2.69829500	-1.49283800	-1.26152900
H	2.63255800	-0.89057200	-2.17272300
H	3.66198800	-2.01130500	-1.27124300
H	1.90272300	-2.23495400	-1.27473000
C	-3.81404200	0.36088700	-0.01536100
H	-3.82940000	0.99319500	-0.90772000
H	-4.73443500	-0.22827100	-0.01574400
H	-3.83887900	1.00668300	0.86705600
C	-2.71079500	-1.47042900	1.27232800
H	-1.91551300	-2.21222100	1.30569300
H	-2.65362300	-0.85284100	2.17379200
H	-3.67467000	-1.98857100	1.28160800
C	-2.69916500	-1.49205000	-1.26150700
H	-3.66321900	-2.00984500	-1.27125900
H	-2.63296100	-0.88985700	-2.17271700
H	-1.90409400	-2.23469300	-1.27459600
O	-0.00028100	-1.83088400	0.02085500

11 H = -735.096534 a.u.

C	-0.30566000	1.97855400	0.00003000
C	1.00506000	1.51167000	0.00014000
C	1.29745400	0.14883100	0.00013800
C	0.20604100	-0.75288100	0.00025300
C	-1.13072500	-0.31016600	0.00001300
C	-1.35978100	1.07234600	-0.00008600
H	1.78797300	2.25538500	0.00010000

H	-2.37424000	1.43642100	-0.00029800
C	2.77146200	-0.32950200	-0.00012500
C	-2.31908600	-1.30124600	-0.00006700
C	-2.27755200	-2.18658600	-1.26934300
H	-1.36396300	-2.77514700	-1.32009200
H	-3.13075100	-2.87253400	-1.27146200
H	-2.34137700	-1.56846900	-2.16973400
C	-3.67944800	-0.57413200	-0.00049700
H	-3.81445300	0.05017100	0.88715000
H	-3.81399600	0.04993700	-0.88837500
H	-4.47975700	-1.31876400	-0.00059700
C	-2.27807300	-2.18611400	1.26954600
H	-2.34225500	-1.56767300	2.16968800
H	-3.13126800	-2.87207100	1.27158300
H	-1.36450400	-2.77465900	1.32083900
C	3.76058500	0.85383000	-0.00077700
H	3.64392500	1.48179600	0.88529300
H	4.78308800	0.46727600	-0.00116100
H	3.64309500	1.48139100	-0.88701300
C	3.08173500	-1.15171200	-1.27947200
H	2.45924200	-2.04016800	-1.40914200
H	2.93010700	-0.53327000	-2.16749800
H	4.12435100	-1.48296900	-1.26951200
C	3.08261800	-1.15085400	1.27958500
H	4.12536700	-1.48167100	1.26936900
H	2.93101500	-0.53204600	2.16735500
H	2.46081700	-2.03971000	1.41022300
O	0.39871600	-2.12174500	0.00026000
H	1.33734100	-2.31540500	0.00360700
O	-0.44698500	3.34322700	0.00006100
C	-1.75677500	3.88442300	0.00011400
H	-2.31895500	3.58737600	0.89357300
H	-1.63283000	4.96660000	0.00023800
H	-2.31895000	3.58758300	-0.89341800

11• H = -734.478788 a.u.

C	0.27877800	1.95629400	-0.00003900
C	1.36354300	1.06406800	-0.00001100
C	1.15960300	-0.30200900	0.00003500
C	-0.22097500	-0.81813900	0.00008200
C	-1.34040900	0.14487900	0.00000000
C	-1.05147000	1.48495800	-0.00007500
H	2.36826400	1.45747100	-0.00011300
H	-1.82692400	2.23853600	-0.00012400
C	2.33772500	-1.28832800	-0.00001500
C	-2.78719500	-0.37038400	-0.00000400
C	-3.04118000	-1.22477500	-1.26697600
H	-2.38621000	-2.09333900	-1.29388900
H	-4.08009700	-1.56899800	-1.27506500
H	-2.87751300	-0.63281000	-2.17264700
C	-3.80649900	0.78467400	-0.00008300

H	-3.71157700	1.41700800	0.88724200
H	-3.71136900	1.41707700	-0.88733900
H	-4.81728000	0.36919300	-0.00022400
C	-3.04128100	-1.22467100	1.26702200
H	-2.87771900	-0.63261400	2.17265300
H	-4.08019200	-1.56891300	1.27503800
H	-2.38629300	-2.09321400	1.29408800
C	3.69922600	-0.56599800	-0.00009400
H	3.83617000	0.05778300	0.88823800
H	4.49817700	-1.31159400	-0.00013100
H	3.83606400	0.05776000	-0.88846300
C	2.27746900	-2.17816000	-1.26699800
H	1.35986200	-2.76194900	-1.29111100
H	2.32982900	-1.56697500	-2.17314300
H	3.13077400	-2.86338200	-1.27451000
C	2.27756800	-2.17811800	1.26697900
H	3.13083200	-2.86338900	1.27441600
H	2.33006500	-1.56691800	2.17311300
H	1.35992700	-2.76185500	1.29121600
O	-0.43981900	-2.05033700	0.00014800
O	0.40122800	3.30238600	-0.00012100
C	1.69952900	3.89282500	0.00015500
H	2.26320400	3.61224600	-0.89470900
H	1.53116500	4.96780700	0.00029000
H	2.26296500	3.61196400	0.89508500

12 H = -694.718583 a.u.

C	1.30164500	1.38154900	-0.02555800
C	-0.02067200	0.91877000	-0.12690300
C	-0.27269400	-0.45835000	-0.13721300
C	2.09163100	-0.93450200	-0.00870200
C	2.33655000	0.44667100	0.02919500
C	-2.67633100	-0.11683500	0.06361000
C	-2.46965000	1.22174100	-0.65471000
H	-3.32666600	1.87746300	-0.47462700
H	-2.43629400	1.01990500	-1.73018400
C	-1.16968100	1.90042700	-0.21754000
H	-0.92269400	2.69894300	-0.92341300
H	-1.31044700	2.39791000	0.75064500
C	1.60923900	2.85910800	0.01743800
H	1.38076700	3.34451700	-0.93836000
H	1.01735600	3.36825200	0.78430900
H	2.66230000	3.02767700	0.23173300
C	3.27307200	-1.87837500	0.03454200
H	2.97580600	-2.91544300	-0.10407800
H	3.99737400	-1.64945700	-0.75720700
H	3.80446300	-1.82496200	0.99405600
C	-3.88339800	-0.86292300	-0.49988800
H	-3.97115400	-1.84809800	-0.03615000
H	-4.80342500	-0.30527700	-0.30638700
H	-3.77640300	-0.99996300	-1.57809400

C	-2.79649400	0.04109400	1.58542300
H	-3.63754400	0.69258800	1.83945700
H	-2.96315200	-0.93431400	2.04787500
H	-1.88974700	0.46756300	2.01783000
O	3.62339400	0.93818900	0.11329700
H	4.23682100	0.20543600	0.21284600
O	-1.54846400	-0.97572500	-0.23475300
C	0.77102900	-1.39854700	-0.07905800
C	0.45010100	-2.87618000	-0.10198100
H	0.81822100	-3.35331700	-1.01678400
H	0.90382700	-3.39978700	0.74481500
H	-0.62492200	-3.03122600	-0.05856900

12● *H* = -694.097226 a.u.

C	1.31954800	1.42234600	-0.01707100
C	0.01699700	0.96242800	-0.09423200
C	-0.21833100	-0.43244500	-0.09378900
C	2.13937400	-0.95546300	-0.00353900
C	2.43455500	0.47989000	0.03485300
C	-2.64212000	-0.12516300	0.04898600
C	-2.41953200	1.20432400	-0.67675500
H	-3.29833000	1.84167400	-0.54503400
H	-2.33091000	0.99470200	-1.74772700
C	-1.15605600	1.91435900	-0.18635900
H	-0.91211600	2.73491600	-0.86623300
H	-1.33573300	2.38209300	0.78934900
C	1.64665400	2.88862100	0.00665500
H	1.36965300	3.37658400	-0.93530700
H	1.10970100	3.40913000	0.80650600
H	2.71706700	3.01718600	0.15525700
C	3.31687500	-1.89307600	0.03771900
H	3.27853200	-2.54991700	0.91266800
H	3.35220600	-2.53377900	-0.84905900
H	4.23580700	-1.31178300	0.08272700
C	-3.77954100	-0.92264200	-0.58234900
H	-3.86744300	-1.90196400	-0.10727700
H	-4.72714100	-0.39190200	-0.46144700
H	-3.59663000	-1.07229600	-1.64844100
C	-2.86108500	0.04560500	1.55630900
H	-3.74451100	0.66193500	1.74366000
H	-3.01486300	-0.92918400	2.02359200
H	-2.00397500	0.51927000	2.03789300
O	3.61431700	0.89201500	0.10458600
O	-1.46213100	-0.96785700	-0.14033200
C	0.83471900	-1.39308600	-0.06479000
C	0.45513600	-2.85338300	-0.09676300
H	1.33122000	-3.49723000	-0.05319900
H	-0.20037800	-3.10455900	0.74179000
H	-0.10281400	-3.09202800	-1.00700000
